# Review of Major and Minor Pathogens of Adult Pacific Salmon (*Oncorhynchus* spp.) in Freshwater in the Pacific Northwest of North America

**DOI:** 10.3390/pathogens15010087

**Published:** 2026-01-13

**Authors:** Tamsen M. Polley, Jayde A. Ferguson, Nora Hickey, Simon R. M. Jones, Anindo Choudhury, John S. Foott, Michael L. Kent

**Affiliations:** 1 Department of Biomedical Sciences and Oregon Veterinary Diagnostic Laboratory, Oregon State University, Corvallis, OR 97330, USA; michael.kent@oregonstate.edu; 2 Wyoming State Veterinary Laboratory, Department of Veterinary Sciences, University of Wyoming, Laramie, WY 82072, USA; 3 Fish Pathology Section, Division of Commercial Fisheries, Alaska Department of Fish & Game, Anchorage, AK 99518, USA; jayde.ferguson@alaska.gov; 4 Washington Animal Disease Diagnostic Laboratory, College of Veterinary Medicine, Washington State University, Pullman, WA 99164, USA; nora.hickey@wsu.edu; 5 Pacific Biological Station, Fisheries and Oceans Canada, Nanaimo, BC V9T 6N7, Canada; simon.jones@dfo-mpo.gc.ca; 6 Division of Health and Human Development, St. Norbert College, De Pere, WI 54115, USA; anindo.choudhury@snc.edu; 7 California-Nevada Fish Health Center, U.S. Fish and Wildlife Service, Anderson, CA 96007, USA; jsfoott@gmail.com; 8 Department of Microbiology, Oregon State University, Corvallis, OR 97330, USA

**Keywords:** Pacific salmon, pathogens, bacteria, viruses, parasites, adult

## Abstract

This comprehensive review examines pathogens affecting adult anadromous Pacific salmon (*Oncorhynchus* spp.) during their terminal freshwater migration and spawning across populations from California through Alaska, including Oregon, Washington, and British Columbia. We systematically reviewed selected pathogens based on their significance to adult salmon health or role in epizootiology, categorizing them by their impact on prespawn mortality (PSM), disease severity, and maternal or ‘egg-associated’ transmission risks to progeny. Our analysis encompasses macroparasites, microparasites, bacteria, and viruses affecting anadromous Pink (*O. gorbuscha*), Chum (*O. keta*), Coho (*O. kisutch*), Sockeye (*O. nerka*), and Chinook Salmon (*O. tshawytscha*) and Steelhead Trout (*O. mykiss*), integrating extensive literature analysis with direct field observations and case studies from representative geographic regions. Understanding pathogen impacts during the spawning life stage is crucial for salmon population sustainability, as the unique semelparous nature of Pacific salmon makes this terminal phase critical for reproductive success and the continuation of these ecologically, economically, and culturally vital species.

## 1. Introduction

Pacific salmon (*Oncorhynchus* spp.) are cornerstone species in the Pacific Northwest (PNW), including Alaska, critical to the region’s ecological, economic, and cultural foundation. A small selection of the many examples of the latter include Pacific salmon providing nutrients to maintain healthy ecosystems and biodiversity [1], native subsistence harvest (valued at $137–275 million annually in Alaska alone [2]), salmon in religious practices by native tribal groups including the “first salmon ceremony” [3], and the Pacific salmon industry accounting for nearly $560,000 of the ex-vessel value of Alaskan harvest while fishing employs nearly one seventh of the Alaskan workforce [4]. Pacific salmon populations face unprecedented challenges from a convergence of environmental and human-induced stressors, including climate change, habitat loss, overharvest, changes in available prey species, predation, novel toxins (e.g., urban runoff) [5], poor ocean conditions, and migratory barriers. The natural reproduction of some populations of Pacific salmon species is enhanced or conserved using hatcheries or spawning channels. However, the unique biological characteristics of these fishes, particularly their anadromous and semelparous life cycle, where they return to freshwater to spawn and subsequently die, make them uniquely sensitive to these extraordinary challenges during their terminal migration and spawning life stage.

As salmon complete this arduous journey, pre-spawning mortality (PSM) emerges as a critical concern for both adult salmon in rivers or streams, or those captured and held in hatcheries before spawning, where sexually mature fish succumb to various intrinsic and extrinsic pressures before completing their reproductive cycle. The multifactorial nature of PSM reveals a complex interplay of stressors, ranging from anthropogenic disruptions to environmental challenges, including river and stream temperatures [6,7,8], stocking density [7], genetic factors, fish length [8], pathogen burden, and disease [9,10] that may all interplay. Underlying these diverse contributors lies a fundamental physiologic transformation. Specifically, adult salmon enter a severe catabolic state due to anorexia and immune suppression from dramatic elevations in cortisol [11,12]. This state creates a critical window of vulnerability, dramatically increasing the risk of opportunistic infections or activation of latent infections. Hence, many pathogens and diseases have been documented in these fishes after they return to freshwater as adults. Several have been directly or indirectly associated with PSM [9,10,13,14,15].

However, many of these infections do not kill fish before they spawn, which is usually in early summer to early winter. As these fish are semelparous, these types of infections may be less consequential than those that kill juvenile salmon, as they are destined to perish. However, these infections can be exacerbated by poor environmental conditions associated with climate change or other stressors, resulting in significant PSM in some systems periodically (e.g., [15,16,17]. For example, increased water temperatures, which can approach the lethal threshold of salmon, increases both fish stress and reproductive rates of their pathogens. Furthermore, some of these pathogens are still of concern when the infected adults, either live or carcasses, are reservoirs for infections to other fish, particularly subsequent generations. Various modes of transmission occur depending on the pathogen, which are discussed in more detail in the following sections. For example, vertical transmission occurs by both direct horizontal or intra-ovum for some pathogens, such as with *Renibacterium salmoninarum*. This bacterium also is associated with PSM and significant mortality of juveniles. Whereas infectious hematopoietic necrosis virus (IHNV) is not associated with PSM, it is a Major concern because it undergoes maternal transmission by extra-ovum mechanisms that causes outbreaks of high mortality of subsequent juvenile populations thereby impacting recruitment, so this is included due to this epizootiological standpoint. Additionally, there is strong evidence that IHNV can also be vertically transmitted in some cases by an intra-ovum route [18,19,20]. Direct horizontal transmission routes also pose substantial risks with several pathogens. These include transmission from antemortem and post-mortem adults to juveniles or to other adults during migration, spawning or as post-spawned live or dead fish. The immunocompromised state of adult spawning salmon [11,12] further exacerbates these transmission risks, as their reduced immune capabilities may allow for increased pathogen shedding and environmental contamination.

This review examines selected pathogens affecting adult Pacific salmon during their freshwater spawning life stage, specifically focusing on populations from California through Alaska, including Oregon and Washington in the United States and British Columbia in Canada. Pathogens of Pacific salmon species covered include those of *Oncorhynchus gorbuscha* (Pink Salmon), *O. keta* (Chum Salmon), *O. kisutch* (Coho Salmon), *O. nerka* (Sockeye Salmon), *O. tshawytscha* (Chinook Salmon), and *O. mykiss* if anadromous (Steelhead Trout).

The list of pathogens, particularly lead by parasites, is enormous and we provide this more complete list as an online repository—i.e., our working database that we used to select pathogens for review (https://ir.library.oregonstate.edu/concern/datasets/st74d079z [accessed on 10 January 2026]). Therefore, we systematically review selected pathogens that we assign to three categories: Major or Minor concerns and Others of interest. For **Major**, we include pathogens that are common and associated with significant disease and PSM, or a risk of parental transmission to progeny that may cause significant mortality. Where significant disease includes a major risk of morbidity, including high rate of spread, or mortality. **Minor** designation includes pathogens of more limited geographic distribution, frequency, or less severe disease or histologic changes. We also include in the Minor category selected previously unpublished observations or unique infection presentations and pathogens of human health concern (e.g., anisakine nematodes). A third category of **Others of interest** includes viral pathogens that do not cause disease or mortality in adult salmon and are not maternally transmitted but can cause significant disease and mortality in juveniles. These viral agents are commonly isolated from apparently healthy adults and have recently been controversial for adult salmon health, thus warranting inclusion to clarify this misunderstanding. Especially due to the challenges of determining the role of incidental infections in moribund fish due to different primary causes when involving previously undescribed infectious agents. This is further complicated by the compromised immune state of these adult fish, multiple pathogens being involved and challenging environmental conditions that continue to change while these fish also undergo programed senescence.

Pathogens are organized by taxa: macroparasites (helminths and arthropods), microparasites (including protozoa, Myxozoa, Oomycota, Ichthyosporea, and Microsporidia), bacteria, and viruses. Notably, viruses in large have not caused measurable mortality of adult Pacific salmon. Pathogens excluded from this review, which are almost all macroparasites, are those which have not been associated with significant lesions, morbidity, or other impacts in adult salmon. Our review explores disease transmission and carrier status within the unique context of salmon’s terminal freshwater phase of life, with particular emphasis on understanding infection opportunities during their critical spawning migration. We aim to provide a comprehensive synthesis of current knowledge about adult salmon pathologic changes associated with pathogens during their freshwater spawning journey. Specific topics addressed include morbidity and mortality patterns, pathologic presentation, modes of transmission, reservoir hosts, and strategies for diagnostics, treatment, and control. Regarding diagnostics, we often cite the diagnostic methods of the American Fisheries Society—Fish Health Section, 2020 edition, “Blue Book: Suggested Procedures for the Detection and Identification of Certain Finfish and Shellfish Pathogens” (https://units.fisheries.org/fhs/fish-health-section-blue-book-2020/ [accessed on 10 January 2026]).

The authors are from representative geographic areas and have had extensive direct experience investigating diseases and PSM in adult salmon. Therefore, in addition to extensive literature review, we include personal observations from our case studies, outbreak investigations, and emerging research findings. We also enlisted advice for our review from other senior fish health experts in the PNW and Alaska, which contributed based on each having several decades of experience with this subject (see Acknowledgements).

**Databases.** Peer-reviewed literature, technical reports, and parasite monographs [21,22] provide background for our review of parasites. For Oregon, we review results from a long-term histologic survey of adult Chinook Salmon in the Willamette System from 2009–2013 and 2017–2023, representing about 1000 fish led by one of us (M.K.).

For Alaska, the disease history database maintained by the Alaska Department of Fish & Game since 1973 was used by one of us (J.F.) to summarize diagnostic cases of wild and hatchery adult salmon with clinical disease or mortality. However, only records dating back to the mid 1980s and 1990s were most complete, so this was the earliest period used for summarizing results. This database also includes results from the National Wildfish Health Survey (NWHS) (https://www.fws.gov/project/national-wild-fish-health-survey-data [accessed on 10 January 2026]). There were 105 cases for Chinook Salmon, none for anadromous Steelhead Trout, 54 for Coho Salmon, 21 for Chum Salmon, 71 for Sockeye Salmon, and 17 cases for Pink Salmon. Regarding broodstock screenings, there were 364 cases for Chinook Salmon, 3 for Steelhead, 408 for Coho Salmon, 85 for Chum Salmon, 201 for Sockeye Salmon, and 84 cases for Pink Salmon.

For Washington State, a repository for diagnostic cases of wild and hatchery fish with 1909 cases for Chinook Salmon, 1175 cases for Rainbow and Steelhead Trout, 94 cases for Coho Salmon, 606 cases for Chum Salmon, 432 cases for Sockeye Salmon, and 51 cases for Pink Salmon were considered.

For California, the NWHS site contains records for most wild fish cases. Hatchery data are kept on an internal Laboratory Information Management System (since 2020) and an access database going back to 1998 (U.S. Fish & Wildlife Service, National Wild Fish Health Survey Data).

**Figure 1:**
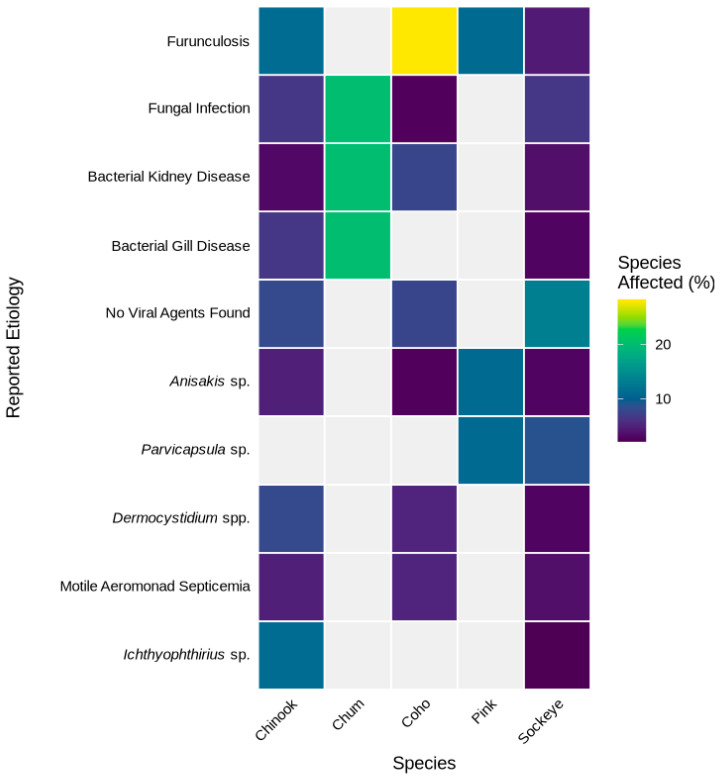
Heatmap of the top 10 reported etiologies in morbidity events among wild adult Pacific salmon occurring in freshwater from the British Columbia Fish Health Database. Data are based on 260 cases between 1973 and 2016 among *Oncorhynchus nerka* (*n* = 136), *O. tshawytscha* (*n* = 62), *O. kisutch* (*n* = 39), *O. gorbuscha* (*n* = 18), and *O. keta* (*n* = 5), with a total of 49 primary etiologies reported. The heatmap displays the percentage of individuals within each species affected by various etiologies. Color intensity represents the proportion (%) of each species population affected, with darker purple indicating low prevalence and chartreuse to yellow indicating high prevalence (viridis color scale). Light gray boxes indicate species-pathogen combinations where no cases were observed.

For British Columbia, in addition to our personal experience (particularly S.J.), we reviewed the British Columbia Fish Health Database (BCFHD), created by the Aquatic Animal Health Section at the Pacific Biological Station, Fisheries and Oceans Canada, Nanaimo, British Columbia. One of us (S.J.) searched the database using adult *Oncorhynchus* species (Chinook, Coho, Sockeye, Chum and Pink Salmon), Life Stage designation (adult, immature adult, ripe adult, jack, spent adult), and “losses” as the criterion for Reason (i.e., reason fish were examined). This resulted in 261 cases in which the primary pathogen observed was listed from 1973–2016. Most of these cases did not include extensive histopathology and only list one etiology, hence we cannot exclude that other etiologies, including other pathogens, were not involved in these PSM events. These data are summarized by the top ten pathogens reported for each species (Figure 1).

Regarding IHNV data, the Molecular Epidemiology of Aquatic Pathogens IHNV database (https://gis.nacse.org/ihnv, accessed on 10 January 2026) was included in addition to the aforementioned resources. This database includes over 3000 records of IHNV, including genetic sequencing, from Western North America from 1966 to present.

We provide selected photographs. Given the long list of pathogens and associated lesions that are discussed, for the most part we restricted our selection of images to demonstrate information that has not been previously published. We include several macroscopic images, which are often not published in peer-reviewed literature.

## 2. Viruses

**Table 1:** Infectious Hematopoietic Necrosis Virus (IHNV) isolated from asymptomatic adult Pacific salmon (*Oncorhynchus* spp.) in freshwater from California to Alaska and summary of juvenile morbidity and mortality patterns.

	Genogroup or Subgroup	Primary Host Species	Other Species Affected	Source (F/M)	System Affected	Disease Description (Juveniles)	Location (State or Province)
	All adult Pacific salmon are asymptomatic carriers of IHNV with risk of maternal transmission.						
**MAJOR**	UP	SO	CH ^a^, CU ^a^, SH, CO ^b^, PI ^b^	F, M	I, Gi, N, Sys (H)	SO major	OR, WA, BC, AK
	UC	CH, SH	SO, CU, CO			CH and ST minor	WA, OR, ID
	M	SH	SO ^b^, CH ^b^, CU, CO			SH major	WA, OR, ID
	L	CH	SH ^b^, CO			CH major	CA, OR

Salmon species: Sockeye (SO), Chinook (CH), Chum (CU), Coho (CO), Steelhead (SH), Pink (PI). Source of infection: M = marine and/or F = freshwater. System affected: I = integument, N = neural, H = hematopoietic, Gi = gill, Sys = systemic/multiple organs. ^a^ reported high morbidity and mortality of juveniles; asymptomatic carriers in returning adults in this region. ^b^ Transient infections.

We found that viral pathogens essentially do not significantly threaten adult Pacific salmon during their spawning migration. We included case studies that followed appropriate disease validation steps [23] rather than relying on molecular detections of viral nucleic acid from fish or tissues without signs of disease, so our findings are restricted to these more rigorous examinations. The clinical significance of viral infections in spawning salmon is generally low without associated clinical manifestations in adults, despite their potential impact on juveniles. Infectious hematopoietic necrosis virus (IHNV) is the predominant viral pathogen of concern (Table 1) due to its maternal transmission from adults to offspring. Although currently no evidence indicates viruses are of concern for PSM in adult Pacific salmon, new molecular technologies or less common approaches have led to the discovery of previously uncharacterized viruses that could potentially become emergent. Thus, screening for these agents should be included for their potential contributions to pre-spawning morbidity/mortality events and reproductive failure in wild and hatchery populations in the future for comprehensive and effective conservation and management of these ecologically, economically and culturally valuable species.

### 2.1. Major Viruses

#### Infectious Hematopoietic Necrosis Virus

Infectious hematopoietic necrosis virus (IHNV) does not cause disease or PSM in adult Pacific salmon, but it can have devastating impacts on juvenile Sockeye Salmon, Chum Salmon, Chinook Salmon, and Steelhead Trout populations. Infectious hematopoietic necrosis (IHN) is a disease of juvenile salmonids where the concern in adults is the risk of maternal transmission. As a member of the Rhabdoviridae family, genus *Novirhabdovirus*, IHNV exhibits the characteristic bullet-shaped morphology typical of rhabdoviruses, measuring approximately 150–200 nm in length and 75–100 nm in diameter [24]. The virion consists of a helical nucleocapsid enclosed within a lipid envelope studded with glycoprotein spikes that facilitate host cell attachment. The virus is endemic in freshwater systems throughout the PNW of North America where it originated, with its endemic presence documented from Alaska to California and inland to Idaho [25,26]. However, the virus has now spread throughout the Northern Hemisphere [27]. In the native range of the PNW, the virus is divided into three major phylogenetic clades known as genogroups: U—from Oregon through Alaska, mostly infecting and causing significant morbidity and mortality in juvenile Sockeye Salmon, but also high mortality in spillover hosts of juvenile Chum and Chinook Salmon in Alaska as discussed below [28]; M—mostly infecting and causing mortality in juvenile Steelhead and Rainbow Trout (*Oncorhynchus mykiss*) in Oregon, Washington, and Idaho; and L—which causes mortality of juvenile Chinook Salmon in California with some detections in coastal rivers of Southern Oregon [29,30].

The U clade, or genogroup, is further partitioned into two subgroups based on genetic ancestry and host-specificity, virulence, and geographic distribution. The UP subgroup is an ancestral virus and a specialist in Sockeye Salmon and rarely infects Chinook Salmon or Steelhead Trout outside the Columbia River Basin in Washington, Oregon, and Idaho. The UC subgroup arose within the Columbia River Basin from a UP ancestor, estimated in the mid-1980s, and has high prevalence in Chinook and Steelhead Trout juveniles without causing substantial disease [31]. One of us (J.F.) has documented outbreaks of the UP genogroup causing high morbidity and mortality in juvenile Chum and Chinook Salmon in Alaska [28,32,33,34,35,36,37,38,39,40,41], whereas Coho and Pink Salmon are refractory to the infection [42]. Several older cases of juvenile Chum Salmon in Alaska experienced acute high mortality of >20% in about 10 days (daily mortality of 3–6%) at salmon enhancement facilities in Southcentral Alaska (J. Ferguson, pers. comm.), prior to the adoption of the Alaska Sockeye Culture Policy [43], which has prevented this from occurring since it was established. Sockeye Salmon can still be infected with the UC subgroup but with slightly lower efficiency and virulence (G. Kurath, pers. comm.). The M genogroup contains four main subgroups, not covered in this review, with mortality occurring in juvenile Steelhead and Rainbow Trout within the Columbia River Basin of Oregon, Washington, and Idaho and sporadically in the Washington Coast [44,45]. For the L genogroup, outbreaks in California in hatchery run juvenile Chinook Salmon are most often detected in mid- to late-run salmon (W. Cox, pers. comm.).

Regarding IHNV in adult Pacific salmon, persistent infections are asymptomatic and this carrier state stands in stark contrast to the disease presentation in juvenile fish, where IHNV triggers extensive destruction of hematopoietic tissues in the kidney and spleen, leading to high mortality rates often up to 100% in susceptible populations, such as Sockeye Salmon [44,46]. In juveniles, the virus replicates rapidly in hematopoietic and epithelial tissues, resulting in characteristic and non-specific clinical signs including whirling, pre-emergence of alevin, exophthalmia, darkened skin coloration, gill hyperplasia, visceral and gill pallor, anemia, necrotic blood cells (necrobiotic bodies), petechial hemorrhages, fecal casts, and abdominal distension. The striking difference in disease expression between adult and juvenile life stages likely stems from age-dependent variations in immune function and tissue vulnerability, with mature fish having developed sufficient innate immunity or immunological memory to suppress viral replication without developing overt disease [24,26,47].

Transmission of IHNV from adult spawning salmon to their offspring occurs through two primary routes. Direct (horizontal) transmission via waterborne exposure represents the dominant pathway, with infected adults shedding virus in urine, feces, and mucus during spawning [26,47]. Indirect vertical “extra-ovum” transmission also occurs through virus association with eggs and reproductive fluids [26,47], though egg disinfection with iodophor significantly reduces this risk [26,47]. However, there is strong supportive evidence for direct intra-ovum transmission by the U genogroup virus in Sockeye Salmon as juveniles have developed the disease from test-positive parents despite the eggs being disinfected and these fish were held in virus free water [18,20]. Adult Sockeye Salmon often harbor IHNV during their spawning migration [47], with virus detection rates and titers increasing as fish approach spawning grounds [26]. This pattern suggests that environmental stressors associated with spawning may trigger viral recrudescence and shedding in previously infected adults. In Alaska, IHNV has also been isolated from asymptomatic adult Chum [48,49,50,51,52,53,54,55,56], Chinook [57], Coho [58,59], and Pink Salmon [60,61] and Steelhead Trout [62] that likely occurred as spillover events when interacting with sympatric Sockeye Salmon stocks where they could act as carriers. Although, in Oregon, previously infected juvenile Chinook Salmon that recovered from IHNV infection of an unknown clade were tagged and released, and upon returning to spawn, no IHNV infection was detected (R. Holt, Oregon Department of Fish & Wildlife (ODF&W), pers. comm.). Genetic analyses have confirmed links between virus strains in adults and subsequent outbreaks in juveniles, highlighting the critical role of adult carriers in maintaining IHNV in salmon populations [18,63]. Some researchers suggest that in Sockeye Salmon, the virus may persist in neural tissues [64]. Environmental factors, including water temperature and quality, influence viral replication, persistence and transmission efficiency in natural and hatchery settings [26,43,47,65].

IHNV screening in adult spawning salmon is a routine component of broodstock management in hatcheries. Standard screening protocols involve sampling kidney-spleen tissue and ovarian fluid from spawning adults, with virus isolation in cell culture on the Epithelioma Papulosum Cyprini (EPC) cell line remaining the gold standard diagnostic method for screening apparently healthy populations, which is also used for diagnostic cases in juveniles [26,47,66]. Chinook Salmon Embryo (CHSE-214) cells have also been reportedly used for virus isolation but are not recommended as they are less susceptible to IHNV infection. For confirmation, molecular diagnostic approaches, particularly reverse transcription polymerase chain reaction (PCR) and its quantitative variants, offer rapid and sensitive detection of viral genetic material [67,68]. When testing large numbers of fish or small fish like alevin, pooled samples are more practical, cost-effective and efficient. Positive detections may trigger management responses including destruction of eggs from infected parents in some regions and often implementation of enhanced biosecurity measures, as no effective therapeutic interventions exist for IHNV infections in adult or juvenile salmon. Alaska has a stringent policy where juveniles with IHN dictates complete destruction of fish within that holding unit. Securing water supplies against contamination from infected wild spawners represents another critical control point, particularly in facilities where adult salmon may be present in upstream waters [24,26,43,44,65]. This systematic screening approach has become a cornerstone of IHNV control in artificial propagation programs [26,44].

Monitoring programs that track IHNV prevalence and concentration in spawning populations provide essential data for risk evaluation and management decisions. Research in Alaska has established that viral titers exceeding 10^4^ plaque-forming units (PFU) per milliliter (mL) in ovarian fluid correlate with increased juvenile mortality risk [69], which could be used to inform thresholds for gamete destruction protocols. Genetic characterization of viral isolates enables tracking of transmission patterns and outbreak sources, as evidenced by studies demonstrating genetic links between virus variants in adults and subsequent infections detected in juvenile populations [63].

Despite extensive investigation, significant questions remain regarding IHNV ecology in spawning salmon. The precise mechanisms and frequency of vertical transmission remain unclear, as does our understanding of how adult carriers maintain and shed the virus over time. The possibility of true intra-ovum vertical transmission, especially in Sockeye Salmon, warrants further exploration, as does the influence of environmental variables on replication and transmission dynamics between adult and juvenile fish [24,26,47]. The role of wild fish and environmental reservoirs in sustaining IHNV cycles is not fully understood, nor are the evolutionary forces driving IHNV adaptation and host range expansion. The potential impacts of climate change on IHNV prevalence, replication and virulence to future progeny in spawning populations represent an emerging concern, with possible implications for transmission risk assessment and management approaches in both artificial and natural spawning environments [63,65,70].

### 2.2. Other Viruses of Interest

These selected viruses are not associated with disease in adult Pacific salmon and have no evidence of maternal transmission. However, they have been isolated in cell culture from routine screening of broodstock by numerous regional or state fish health laboratories. These findings parallel observations in other vertebrates, where viral presence does not necessarily correlate with clinical disease. As Mortimer describes with “orphan viruses,” many viruses can be present asymptomatically or as part of the normal virome, and their detection does not automatically indicate pathogenicity [71]. While IHNV remains the predominant viral pathogen of concern in spawning Pacific salmon due to high mortality of juveniles and evidence of maternal transmission, recent research has identified novel viruses worth mentioning that have either been detected as an incidental finding or by molecular methods. However, like other infections, these may have the potential to be impactful under specific environmental or physiological conditions that may be more problematic in the future.

One example of an incidental finding during routine screening involves a novel Salmon **Flavivirus** (SFV) that was recently isolated from three moribund adult Chinook Salmon in California’s Eel River due to other primary causes. This represents the first characterized aquatic flavivirus in Pacific salmon [72]. The case history involved a high density of adults (~1500) congregating in a small, shallow pool with low water flow associated with recent draught and high algal growth indicative of poor environmental conditions, such as high water temperature, low dissolved oxygen, and high alkalinity, which likely approached the lethal limit for these fish. About 150 of the 1500 adults in this pool appeared moribund (10%) and 20% of these fish had a high infestation of the eye fluke, *Diplostomum* sp., which was associated with ocular opacity and signs of blindness. The primary diagnosis was poor environmental conditions, draught, warm water, algae, and high parasite burden associated with blindness. The algae and low flows likely attracted many snail hosts with the flukes, further reduced the dissolved oxygen, and may have increased the alkalinity. The Eel River fish exhibited non-specific clinical signs including lethargy, abnormal behavior, and petechial hemorrhages on the surface of the brain. Although histopathology of the brain confirmed gross observations of petechial hemorrhages on the optic lobes, cerebellum, and spinal cord, these are non-specific changes that were most likely due to the heavy ocular parasitism or postmortem changes. No other significant microscopic findings were noted in the heart, liver, spleen, kidney, intestine, or skeletal muscle or presence of viral nucleic material by in situ hybridization (ISH). Petechial hemorrhages of the meninges and central nervous system should be interpreted with caution, as they can be incidental postmortem artifacts [73,74,75].

Soto et al. followed with a laboratory infection study using juvenile Rainbow Trout and Chinook Salmon exposed to the viral inoculum by immersion and intraperitoneal injection [72]. This challenge demonstrated that SFV can replicate in Chinook Salmon, particularly in brain tissue, indicating potential for neurotropism. However, despite fulfilling Rivers’ postulates through infection of healthy Chinook Salmon and viral re-isolation, laboratory infections produced no clinical disease, mortality, or microscopic pathology despite molecular evidence of viral replication [72]. This indicates that SFV is not a primary pathogen but may have the potential to be an opportunist under specific conditions or in conjunction with other stressors affecting salmon. One obvious stressor in adult salmon would be immunocompromisation. This is widespread to some degree in all sexually maturing Pacific salmon and can be exacerbated by environmental and anthropogenic stressors. Alternatively, this may represent an orphan virus that is not associated with disease at all.

Another example of a recently described virus without evidence of PSM in adult Pacific salmon or maternal transmission is **piscine orthoreovirus (PRV)**, which has been associated with minimal clinical signs in juvenile Coho Salmon. There have been two strains detected in clinically normal adult Pacific salmon in freshwater in the PNW [76]. PRV-1a is common in seawater populations of Chinook and Coho Salmon in this region [77] and minimally observed in freshwater populations [78], although it has been detected in other *Oncorhynchus* species (reviewed by Polinski et al. [76]). Infection in apparently healthy juvenile and adult fish is also common. Public interest in PRV is high, as this virus is sometimes featured in media coverage speculating the causes of Pacific salmon population declines, but there remains no scientific proof of PRV-impact on Pacific salmon populations [79].

An overwhelming body of evidence, to date, has demonstrated that PRV does not cause disease in adult Pacific Salmon [76]. Multiple challenge trials in laboratories have shown PRV-1a does not cause morbidity or mortality in juvenile Pacific salmon species in both fresh and saltwater [76,80,81,82]. Nevertheless, in juvenile European Atlantic Salmon (*Salmo salar*) where occasional PRV-1 disease causation has been established [83], the pathogenicity has been shown to be complex and multifactorial [84]. However, in the Northeast Pacific, PRV-1 has been unsubstantially speculated to cause jaundice syndrome in juvenile Chinook Salmon [85], yet PRV could not be made to recreate disease in the laboratory [80] and PRV could not be detected in jaundiced Chinook Salmon captive broodstock in Washington State as well as in 2019 wild returning jaundiced Pink Salmon in British Columbia (N. Hickey and M. Polinski, pers. comm.). PRV-2 has been associated with erythrocytic inclusion body syndrome (EIBS) in Coho Salmon from Japan [86], and a PRV-2-like virus was detected in diseased freshwater juvenile Coho Salmon associated with anemia and chronic low-level mortality in Alaska in 2021 [87]. EIBS also occurs in salmon from the PNW, but the virus causing these inclusions has not been adequately described [88,89,90].

**Viral erythrocytic necrosis virus** (VENV) is a marine virus that infects a diversity of fish families but is most pathogenic in Pacific Herring (*Clupea pallasii*) [91]. It has been diagnosed from blood smears in the marine phase of all five Pacific salmon species [92,93]. Experimental infections have shown that juvenile Pink and Chum Salmon to be the most susceptible whereas Chinook, Coho, and Sockeye Salmon are more resistant [92,94,95]. There this no evidence of maternal transmission for this virus.

**Infectious pancreatic necrosis virus** (IPNV) is cosmopolitan and is a serious pathogen of juvenile Pacific salmon but does not cause morbidity or mortality in pre-spawning adults and lacks evidence for maternal transmission. It was once detected in spawning adult Coho Salmon at Bonneville Hatchery in Oregon in 1971 (R. Holt, ODF&W, pers. comm.) where it was also seen in their progeny. This virus has also been detected during routine surveillance of broodstock in Washington State (N. Hickey, pers. comm.). This report of IPNV represents a common finding with many salmonid viruses, such as IHNV, in which they can be pathogenic in juveniles, but their effects in adults are absent.

Other viruses that are not reviewed here are not recognized as pathogens of either adult or juvenile Pacific salmon in freshwater (i.e., orphan viruses) and have no evidence of maternal transmission and are thus regarded as not of concern. These are commonly isolated from apparently healthy broodstock as part of surveillance programs. Examples include viral hemorrhagic septicemia virus (VHSV) genotype IVa [96,97], fall Chinook aquareovirus [98], cutthroat trout virus (CTV-1 and CTV-2) [99,100], and Pacific salmon paramyxovirus type A [101].

## 3. Bacteria

**Table 2:** Bacterial pathogens in adult Pacific Salmon (*Oncorhynchus* spp.) in freshwater from California to Alaska. Major: significant disease or other concerns, and widespread. Minor: restricted outbreaks, although they may cause severe lesions or morbidity.

	Bacteria	Source (F/M)	System Affected	Disease Description	Location (State or Province)	MT
	All salmon species are affected (Sockeye, Chinook, Chum, Coho, Steelhead, and Pink Salmon).					
**MAJOR**	*Aeromonas salmonicida* (Furunculosis)	F	I, R, M, S, Sys	No signs, acute mortality, necrohemorrhagic “furuncles”, splenomegaly, renal necrosis	worldwide	
	Columnaris-causing bacteria (Columnaris Disease)	F	I, Gi	Gill necrosis, skin/fin ulceration and necrosis	worldwide	
	*Renibacterium salmoninarum* (Bacterial Kidney Disease)	F	R, S, M, Sys	Exophthalmia, skin petechia, ascites, pericardial effusion, granulomas, splenomegaly	CA, WA, OR, BC, AK	✓ *
**MINOR**	*Flavobacterium psychrophilum* (Bacterial Coldwater Disease)	F	I, R, M, N, S, Gi	Disease in juveniles: Fin and caudal peduncle necrosis, neurologic signs	CA, OR, WA, BC, AK	✓ *
	*Mycobacterium salmoniphilum* (Mycobacteriosis)	F, M	Sys	Granulomas	worldwide	
	*Piscirickettsia salmonis* (Salmonid Rickettsial Septicemia)	F, M	Sys	Systemic, renomegaly, splenomegaly, ascites, exophthalmia, sepsis	CA, WA, OR, BC	

Source of infection: M = marine and/or F = freshwater. MT = maternal or ‘egg-associated’ transmission (including direct vertical via intra-ovum and indirect transmission through contaminated tissues/fluids). System affected: I = integument, R = renal, M = musculoskeletal, N = neural, S = spleen, Gi = gill, Sys = systemic. * Risk of life-long carrier status after recovery from infection. ✓ The check mark confirms maternal transmission (MT) with the respective pathogen.

Bacterial diseases represent a significant threat to spawning Pacific salmon, often exacerbated by physiological stress, immunocompromised status, and the physical demands and trauma of upstream migration. This is further exacerbated by environmental stressors, such as elevated water temperatures [8,9,102,103] or low dissolved oxygen. Several major bacterial pathogens, including *Aeromonas salmonicida* (Furunculosis), *Flavobacterium columnare* and other *Flavobacterium* spp. (Columnaris Disease) and *Renibacterium salmoninarum* (Bacterial Kidney Disease), in addition to other minor bacterial pathogens, can severely impact spawning success and survival (Table 2). These bacterial pathogens fall into two broad categories: obligate (primary) pathogens that cause disease regardless of host condition, and opportunistic (secondary) pathogens that typically cause disease only in compromised hosts. This distinction determines the management approach to disease outbreaks. Management of these bacterial infections is also critical due to the risk of transmission to progeny either directly (vertical transmission) for some or indirectly (horizontal transmission) for all.

### 3.1. Major Bacteria

#### 3.1.1. *Aeromonas salmonicida* (Furunculosis)

*Aeromonas salmonicida*, the causal agent of furunculosis, is one of the most serious bacterial diseases of captive-held and wild salmonids representing a major cause of PSM, particularly in returning broodstock held in hatcheries prior to spawning. The bacterium affects all *Oncorhynchus* species throughout the North Pacific and can cause significant mortality during both the juvenile rearing phase and adult spawning migration [104]. While long recognized as a serious problem in juveniles in freshwater hatcheries and seawater net pen-reared Atlantic Salmon, its impact on adult Pacific salmon during spawning migrations is less well documented and may be an emerging concern due to climate change.

In adult Pacific salmon, outbreaks have been documented in spawning adult fish in Alaska and Oregon rivers. Kent et al. found that among fish examined over multiple years, the infection was actually detected more frequently in live than dead fish [105]. Benda et al. reported that the infection was more common in PSM adult Chinook Salmon held in tanks compared to fish from the same population in the river [9], suggesting that captivity may exacerbate disease progression, although wild fish may congregate in high densities and be at similar risks in those situations. Infections associated with PSM in Coho Salmon [106,107,108], Chinook Salmon [109,110,111], and Pink Salmon [112,113] occurred several times in Alaska. The latter cases that involved Pink Salmon broodstock were complicated by environmental stressors and in the more recent case also by gill parasitisms.

*Aeromonas salmonicida* is a Gram-negative rod-shaped bacterium that is beta hemolytic and typically produces a brown diffusible pigment when grown on culture media [114]. Most strains are non-motile, a characteristic that differentiates *A. salmonicida* from other *Aeromonas* species that infect fish [115]. Atypical strains that do not produce brown diffusible pigment are classified as the subspecies *achromogenes* [116].

The namesake “furuncles” are necrohemorrhagic foci within the dermis or deeper tissues that may ulcerate and can extend deep into the skeletal muscle, but these lesions are not consistently present in all clinical cases. In acute cases, fish may die suddenly with few clinical signs, or mortality may be peracute without any signs at all. Typical gross lesions include exophthalmia, splenomegaly, friable kidneys due to renal necrosis, ascites hemorrhage around the fin bases and vent, and petechial hemorrhage on serosal surfaces and in the fillet. A particularly interesting and poorly understood lesion is the development of a grossly apparent blue-green color to the iris [117], most likely due to iris depigmentation of the anterior iris surface. Iris depigmentation in humans has been documented as bilateral acute depigmentation of iris (BADI) and bilateral acute iris transillumination (BAIT) with an unknown etiology, but some theorize associations with viral infections or antibiotic therapy [118,119]. Liquefactive necrosis of the kidney can be severe for fish with furunculosis. Histologically, large aggregates of bacteria can be easily visualized in target organs including the kidney, spleen, and gills, often with little inflammatory reaction, due to defensive mechanisms by the bacterium involving effectors and toxins, but extensive regional necrosis occurs surrounding bacterial colonies from their release of extracellular products, such as proteases and hemolysins.

Transmission occurs primarily horizontally between fish via the water column or fecal-oral route [120], with arthropod parasite vectors likely playing a role. Gill copepods, such as *Salmincola californiensis*, are the most likely candidates in the freshwater phase of Pacific salmonids [117]. The initial site of infection of the bacterium is believed to be the gills. Latent infection is an important aspect of the epizootiology, with chronically infected fish persisting as carriers in a population and contributing to persistent mortality after the acute phase has resolved [121]. Maternal transmission has not been shown to occur for this bacterial pathogen.

Two major environmental factors affect disease development: water temperature and fish density. Mortalities increase as water temperature rises [122,123], suggesting that climate change may make *A. salmonicida* an increasingly prevalent contributor to PSM in Pacific salmon. Mortalities also increase with fish density [123,124], explaining the higher prevalence in captive settings compared to wild populations, but wild fish that congregate in high densities could also be prone to infection.

Diagnosis relies on clinical signs, gross pathology, and bacterial culture, with definitive diagnosis requiring isolation in culture or identification of the bacterium from infected tissues by fluorescent antibody testing (FAT) or PCR. Histopathology can be useful for interpreting bacterial culture results. Antimicrobial susceptibility testing can be performed using minimal inhibitory concentrations and disk diffusion, and clinical breakpoints and epidemiological cutoff values for *A. salmonicida* have been established for several antibiotics commonly used in aquaculture [125]. *A. salmonicida* has been documented to have antimicrobial resistance, and a strain has been isolated from Chinook Salmon in Washington that is resistant to all three antibiotics approved for use in aquaculture in the United States (N. Hickey, pers. comm.).

In juvenile salmonids florfenicol, oxytetracycline, and sulfadimethoxine ormetoprim medicated feeds are approved for treatment of furunculosis. However, medicated feeds are not an option for treating returning broodstock as these fish are not eating during this life stage. In hatchery settings, injectable antibiotics approved for use in other food animals are used extra-label for treating furunculosis in fish, including Liquamycin LA-200 (oxytetracycline; Zoetis; Parsippany, NJ, USA) and Nuflor (florfenicol; Merck; Rahway, NJ, USA) that can be used to control PSM of adults. Establishing withdrawal times for senescent fish is challenging because of their altered metabolic state [126]. Vaccines have been used extensively to protect Atlantic Salmon in seawater netpens and juvenile Pacific salmon in hatcheries.

Management strategies focus on prevention through egg disinfection, keeping water temperatures cool by using cold water sources and shade, reducing fish density, reducing handling trauma and subsequent skin barrier compromise, and preventing secondary diseases such as saprolegniasis. Managing adult salmon with *A. salmonicida* infections is important as the bacterium can be transmitted from adults to juveniles in the same watershed.

Several knowledge gaps remain regarding *A. salmonicida* in adult spawning Pacific salmon: the relative importance of new versus chronic infections in returning adults; treatment efficacy for adults; the quantitative impact of climate change on disease prevalence; the role of specific vectors in natural settings; and the contribution to PSM in wild populations versus hatchery settings. Further research addressing these gaps would improve our understanding of this pathogen’s role in PSM and inform more effective management strategies.

#### 3.1.2. Columnaris-Causing Bacteria (Columnaris Disease)

Columnaris disease, caused by columnaris-causing bacteria including *Flavobacterium columnare*, *F. davisii*, *F. covae*, and *F. oreochromis*, are significant pathogens in both cultured and wild fish populations worldwide [127,128] and is emerging as one of the most important pathogens for PSM in Washington State, which may be linked to warming rivers associated with climate change. *F. columnare* and *F. davisii* have been isolated from Pacific salmon in the PNW [129]. Flavobacteria are thin, filamentous, Gram-negative bacilli (3–10 µm long) with occasional gliding or flexing motility that produce yellow orange pigmented rhizoid colonies on agar. Media for isolation includes selective Sheih’s with tobramycin [130], Hsu-Shotts, and Cytophaga [131]. For molecular detection methods, LaFrentz et al. [132] developed a multiplex PCR that targets the *dnaK* gene for *F. columnare* genetic group identification. In the environment, the bacteria can exist as a sessile saprophyte and infective planktonic form [133].

Virulence is associated with extracellular proteases such as Chondroitin AC lyase [134]. Fish mucus can function as a chemotactic factor as well as enhancing biofilm formation, gill attachment, and extracellular protease production [135,136,137]. Adherence to the gill is enhanced by elevated water temperatures, organic matter, and ion-rich water [138]. *F. columnare* infections in juvenile Steelhead Trout and salmon begin to occur when water temperatures reach 15 °C and become progressively more severe as temperatures increase to 20–24 °C [139].

**Figure 2:**
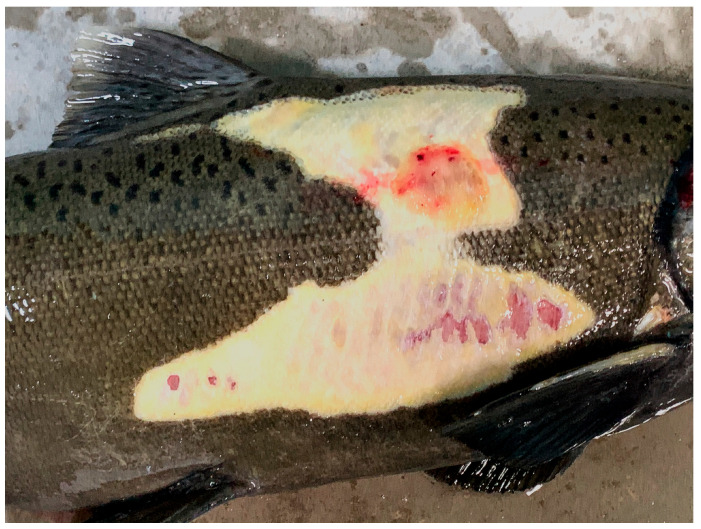
Columnaris disease in a returning adult spring Chinook Salmon. The classic presentation is extensive, pale yellow, necrotic and ulcerative dermatitis, that in advanced disease, can extend into the underlying deep dermis and muscle. These ulcers are most commonly present on the caudal fin, sides, and ventrum of the fish.

Clinical signs of infection include gill necrosis, localized discoloration, erosion, and ulceration of the skin surrounded by erythema, and erosion of fins [127]. In advanced disease severe tissue necrosis into the dermis and underlying muscle is present with a yellow tinge to the tissue (Figure 2). The gill is reported as initial site of colonization and infection [138]. The shedding rate from live infected fish is reported to range from 10^3^–10^5^ colony-forming units (CFU)/mL while carcasses can produce over 10^6^ CFU/mL [133,140]. Increasing water flow decreases transmission efficacy [141,142]. Congregation of fish within thermal refugia has been associated with disease epizootics such as very high mortality in Chinook Salmon in 2002 in the Klamath River, California, along with increased disease transmission [143,144].

Columnaris-causing bacteria have long been known to have an exceptionally high level of genetic diversity, and recent advances in molecular technology have identified four distinct species of bacteria previously classified as *F. columnare* [145,146,147]. As such, the current literature is reclassifying the etiology of Columnaris Disease as “columnaris-causing bacteria” [148]. Future work on the pathogenicity and management of *F. columnare* may require additional work to characterize these genetic groups.

Treatment of adult fish with Columnaris Disease is challenging. Handling and upstream migration can be a source for traumatic skin lesions that are slow to heal and predispose fish to infection by columnaris-causing bacteria. In addition, elevated water temperatures are usually a causal environmental factor in this disease process that may not be possible to address by the facility holding broodstock. Antibiotic injections are often ineffective as well as being a source of additional handling and stress for the fish. External treatments including Reward (diquat; Syngenta; Greensboro, NC, USA), Halamid Aqua (chloramine-T; Syndel; Ferndale, WA, USA), and 35% PEROX-AID (hydrogen peroxide; Syndel; Ferndale, WA, USA) can be highly effective in controlling columnaris disease and preventing PSM in broodstock held at hatcheries. Early application immediately after broodstock collection and before the disease progresses significantly improves treatment efficacy.

Columnaris disease has historically been a problem of warmwater fish species, but it is an emerging cause of PSM in *Oncorhynchus* spp. in the PNW as climate change causes increased temperatures and low flows in certain salmon spawning habitats. Future directions of study include understanding which of the four distinct species of columnaris-causing bacteria infect Pacific salmon and characterizing these infections; the quantitative impacts of increased water temperature on the disease; and exploring external and systemic treatments for adult salmon.

#### 3.1.3. *Renibacterium salmoninarum* (Bacterial Kidney Disease)

*Renibacterium salmoninarum* is the causal agent of bacterial kidney disease (BKD) in salmonids. *R. salmoninarum* is a common infection of returning broodstock and can cause disease and PSM in Chinook and Sockeye Salmon in particular [149,150]. This bacterium infects all *Oncorhynchus* species throughout the North Pacific and can also cause significant mortality during the juvenile rearing phase in fish six months and older [104]. *R. salmoninarum* is of particular concern to salmonid conservation programs because vertical transmission from broodstock to juveniles is a major source of the bacterium for BKD outbreaks during hatchery rearing of the latter. For this reason, many hatchery programs spend significant time and effort managing this bacterium in their broodstock even if it is not causing PSM. Nevertheless, the bacterium was the cause of a high PSM event of adult Sockeye Salmon in Alaska in 2018 involving about 30% of female broodfish holding in a lake resulting in about a 50% reduction in eggs taken that impacted the fisheries in future years of returns [151,152]. BKD has also caused PSM in Chinook Salmon in Oregon [9,149,150].

**Figure 3:**
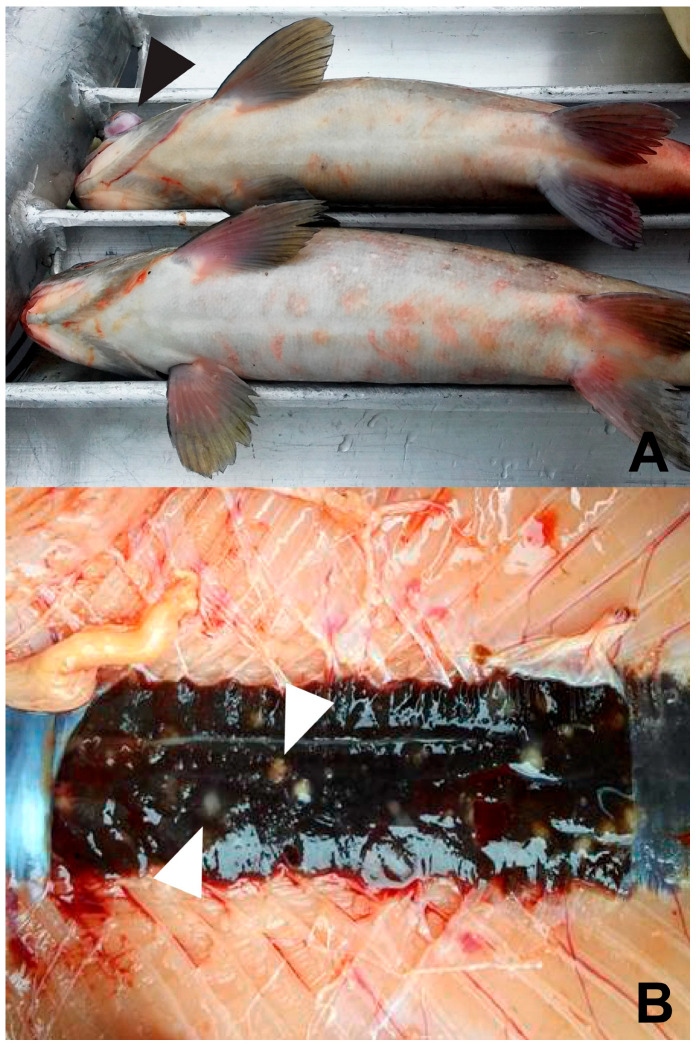
Bacterial kidney disease (BKD) in adult Sockeye Salmon from Bear Lake in Alaska during a BKD epizootic [154]. (**A**) Adult females with external signs of bacterial sepsis including exophthalmia (black arrowhead) and erythema or “spawning rash.” (**B**) Renomegaly with removal of the peritoneum demonstrating multifocal, pale foci throughout the renal parenchyma due to granulomatous inflammation (white arrowheads).

Clinical signs of BKD include exophthalmia, petechial hemorrhages and/or vesicles of the skin, and abdominal distention from abdominal and pericardial ascites (Figure 3A). The disease is usually chronic and involves granulomas or granulomatous inflammation. The kidney is the target organ and is often enlarged and edematous and may exhibit off-white granulomas varying in size (Figure 3B). The whole kidney may appear gray, corrugated, and enlarged. Granulomas may also be present in other organs, such as the liver and spleen. Besides the granulomas, splenomegaly is common and may be one of the first indications of BKD when the coelom is initially opened. An integumentary lesion, called a “spawning rash” in adults, may be seen [153], which is comprised of multifocal, ulcerative lesions with no defined epidermal predilection (Figure 3A). Histologically, granulomas are present in affected organs and bacteria can often be visualized within the cytoplasm of intralesional macrophages.

The chronic nature of this infection is a critical part of the epizootiology, with infected fish becoming carriers for life and returning to their natal streams to infect the next generation. *R. salmoninarum* can also be transmitted horizontally from fish to fish, via water containing infected fish, or via contaminated feed. The organism may survive free in the environment for long periods of time. Additionally, it has now known for decades that the bacterium can also be transmitted vertically within the egg [155]. The bacteria gain access during egg formation or more commonly enter the yolk through the micropyle after ovulation from contaminated ovarian fluids of the female broodfish.

Presumptive diagnosis of BKD can be made by observation of the gross pathologic changes, but differentials based on these clinical signs may include ichthyophoniasis, mycobacteriosis, proliferative kidney disease (PKD), or nephrocalcinosis. The bacterium replicates extracellularly and intracellularly within macrophages, and the detection of Gram-positive, small, non-acid-fast, non-spore forming, non-motile coccobacilli in both locations in impression smears provides a strong presumptive diagnosis. The infection can be confirmed with a specific fluorescent antibody test, enzyme-linked immunoabsorbent assay (ELISA), PCR, or quantitative PCR (qPCR) [153]. Special media and extended incubation times of up to 6 weeks are required for successfully culturing the organism.

There can be significant non-concordance between different testing methodologies and tissues for the same individual fish. Proper interpretation of test results requires understanding what exactly each test is detecting, spanning from culture requiring live bacteria to PCR detecting nucleic acid to ELISA detecting bacterial antigen. To further complicate interpretation, the soluble p57 antigen detected by the ELISA can persist in the tissues of infected fish for months in the absence of live bacteria [156]. While all testing methodologies work well for diagnosing clinical disease, their utility for identifying subclinical infections varies. Screening apparently healthy returning adult salmonids by ELISA and culling eggs from females with higher optical densities is a common strategy for reducing the risk of vertical transmission to hatchery juveniles [157]. However, the ELISA is used on kidney tissues and not ovarian fluid, which is involved in vertical transmission but inhibits detection by ELISA [158].

*R. salmoninarum* commonly infects most salmonids. Where ELISA testing in Alaska has shown that all five Pacific salmon species have tested positive across most watersheds with prevalence ranging from 10–90%. Where trout species (*Oncorhynchus* and *Salvelinus* spp.), Arctic Char (*Salvelinus alpinus*), and Arctic Grayling (*Thymallus arcticus*) are reservoirs for the bacterium with up to 100% prevalence [159] and can exhibit morbidity and mortality.

Antibiotic treatments have poor efficacy in returning adult salmon with BKD due to the chronic and intracellular features of this infection, which cannot be administered by medicated feeds because these fish have ceased eating at this life stage. In hatcheries, injectable antibiotics approved for use in other food animals are used extra-label for treating BKD, including Liquamycin LA-200 (oxytetracycline; Zoetis; NJ, USA) and Draxxin (tulathromycin; Zoetis; NJ, USA), although mixed results have been obtained with the latter. Erythromycin injectable products are not currently approved in the United States. Sometimes imported injectable erythromycin products become available for use as investigational new animal drugs (INADs), but their availability is inconsistent. Toxicity is a common adverse effect of erythromycin treatment in salmonids [160], which include sudden death syndrome in juveniles.

Knowledge gaps that remain regarding *R. salmoninarum* in adult spawning Pacific salmon include: application of non-lethal screening methods to identify adults at high-risk of vertically transmitting *R. salmoninarum* to their offspring; test methods for eggs to determine egg infection status; testing of ovarian fluids instead of kidney for culling eggs in maternal tracking because this may be a more appropriate tissue; and treatment efficacy for adults. Further research in these areas would improve our understanding of this pathogen’s role in PSM and downstream effects of infected adults including vertical transmission to juvenile. This would provide more effective management strategies at multiple life stages of the salmonid rearing cycle.

### 3.2. Minor Bacteria

#### 3.2.1. *Flavobacterium psychrophilum* (Bacterial Coldwater Disease)

Bacterial coldwater disease (BCWD), caused by *Flavobacterium psychrophilum*, does not typically cause primary disease and mortality in adults, but can cause high mortality (e.g., >0.05%/day) of juvenile salmon. It is ubiquitous in the environment, including biofilm formation, where it is transmitted horizontally and can also be vertically transmitted both within eggs and contaminated gametes of infected adult salmonids [161,162,163] where returning broodstocks can act as carriers. Thus, it is included here due to this transmission and potential to cause high mortality of progeny that could impact population recruitment. The bacterium is endemic to the PNW and Alaska, and all salmonids are regarded as susceptible, especially juvenile Coho and Chinook Salmon and Steelhead Trout [164].

It is a Gram-negative, proteolytic bacterium that causes systemic disease in juveniles in colder waters. Clinical signs usually occur below 12 °C, but it has been reported at temperatures as low as 1 °C [165]. The bacterium produces yellow-pigmented colonies that are typically convex or with convex centers and a spreading periphery resembling a “fried egg” appearance. It grows on Cytophaga and Tryptic Yeast Extract Serum (TYES) [166], Hsu-Shotts, *Flavobacterium psychrophilum* medium A (FPM-A) [167], and *Brucella* agars, with optimum growth at 15–16 °C. Notably, *F. psychophilum* does not grow on blood agar. Colonies are comprised of long, filamentous, non-motile Gram-negative bacilli that may rarely display gliding or flexing motility. Colonies turn orange-red when KOH is added indicating flexirubin pigment. Growth of *F. psychrophilum* is inhibited by Congo red added to TYES agar or diffusion disks that may aid in identification, which is non-specific at the species level. However, confirmatory testing is performed by PCR/qPCR or direct FAT.

BCWD often starts as an external infection that becomes systemic, with clinical signs of ascites and hemorrhage. Both infection types can cause fish mortality. Presumptive cases in moribund adult broodstock with elevated mortality involved external flavobacteriosis in Coho Salmon with primary furunculosis [168,169] and the primary cause in Chinook Salmon [170,171]. This bacterium can often be difficult to isolate from clinical cases, so many times the diagnosis is restricted to external and/or systemic *Flavobacterium* sp. External infections can be controlled by administering approved standard external chemotherapy, such as formalin [172]. Typically, antibiotic therapy is not administered to adult salmon due to drug withdrawal requirements and other concerns.

#### 3.2.2. *Mycobacterium salmoniphilum* and *Mycobacterium* spp. (Mycobacteriosis)

Mycobacteriosis is occasionally observed in adult *Oncorhynchus* species [173,174]. It was much more common in the 1950s and 1960s, possibly linked to feeding raw fish products to salmon in hatcheries. Ross provides a thorough review for that time period [175]. More recently, infections have been noted in marine pen-reared Atlantic Salmon, including in the PNW [176,177].

Both *M. salmoniphilum* and *M. chelonae* have been implicated as the causative agent in salmonid mycobacteriosis. Moreover, these species are related and differentiation has been unclear. *M. salmoniarium* was described from salmon by Ross [178]. Later, based on further biochemical characterizations, Arakawa and Fryer concluded it was a subspecies of *M. chelonae* [179]. Then Whipps et al. showed, using molecular methods, that various isolates from adult salmon in the PNW actually formed a distinct clade using hsp65 analysis [180]. Hence, along with distinct phenotypic characters (e.g., inability to grow at 37 °C), they re-established the species *M. salmoninarum*. Added to this confusion, early reports found that mycobacteria were very difficult, if not impossible, to culture from infected adult salmon fish. Hence, some cases linked to feeding raw fish may be caused by a more fastidious organism. For example, with mycobacteriosis in poecilid fishes in Hawaii [181], direct sequence from infected tissue of several fish showed that the most likely cause was a *M. triplex-like* organism, although *M. chelonae* was cultured from a few of these fish.

The pathologic changes, both macroscopic and histologic, are typical of chronic bacterial infections in salmonids and may be confused with BKD [177]. The kidney, and sometimes spleen, are enlarged. Multiple whitish focal lesions may be seen in the infected organs—e.g., kidney and liver. Histologically, loosely organized, diffuse granulomatous lesions are seen, and acid-fast stains reveal numerous bacteria [177]. This is in contrast with *M. marinum* infections, which are often the cause of mycobacteriosis in fishes. The latter causes well organized granulomas comprising epithelioid macrophages, and often acid-fast bacteria are sparse. With *R. salmoniarium* infections, bacteria should not be acid fast positive. Further confirmatory diagnosis can be achieved with specific PCR and immunohistochemistry (IHC) tests [182], and *M. salmoniphilum* and *M. chelonae* are “fast growers” and hence easier to grow than many other *Mycobacterium* spp. of fishes [180]. Organisms in culture can then be identified with molecular methods. Whole genome studies suggested that *M. salmoniphilum* was related to *M. franklinii* [183]. This disease cannot be effectively treated, so culling of diseased fish in cultured populations remains the only method for attempting to control outbreaks.

Many of the *Mycobacterium* species from fishes also infect humans, particularly *M. marinum.* However, *M. salmoniphilum* does not grow at 37 °C [180], and there have been no connections of this bacterium to human infections. *M. chelonae* does infect humans, but we are not aware of any direct links to these infections from fish.

#### 3.2.3. *Piscirickettsia salmonis* (Salmonid Rickettsial Septicemia)

This bacterium is a facultative intracellular, Gram-negative, non-motile cocci-shaped bacterium. It causes piscirickettsiosis or Salmonid Rickettsial Septicemia (SRS), which is a disease of farmed salmonids in saltwater with scant reports of it occurring in freshwater. Infections have been reported from salmonid and non-salmonid fishes with global distribution. Impacts of the disease are greatest in Chile where infections occur in farmed Coho and Chinook Salmon, Rainbow Trout, and Atlantic Salmon with greater than 90% mortality and significant economic impacts [184,185]. Less severe manifestations of piscirickettsiosis occur in farmed Atlantic salmon eastern and western Canada, Norway, Scotland, and Ireland and farmed Chinook and Coho Salmon in western Canada [186,187,188,189,190,191,192]. The disease in farmed juveniles and adults is characterized by swollen and discolored kidneys, splenomegaly, accompanied by ascites, exophthalmia and hemorrhage of skin, organs, skeletal muscle and brain, consistent with septicemia. Bacteria are shed from infected fish in proportion to the severity of the disease, serving as a direct route of transmission [193].

The available evidence suggests infections occur at low prevalence in adult Pacific salmon in freshwater. In British Columbia, evidence of the infection with disease occurred in laboratory-reared juvenile Pink Salmon [186,187,194], however neither the prevalence of the infection nor the origin of the fish was reported. The infection was later reported in 1 of 100 archived kidney samples from apparently healthy spawning Pink Salmon screened by qPCR [195]. Evidence of the infection obtained by using a high-throughput molecular screening tool was observed in fewer than 1% of apparently healthy adult Fraser River Sockeye Salmon [196] and in 11.1% of archived Sockeye Salmon samples collected from various British Columbia watersheds between 1985 and 1994 [197], but not in 85 adult Chinook Salmon [198]. There are few reports of SRS in populations of wild Pacific salmon [199] and the reservoir of infections found in farmed salmon is not known. However, in other regions, it has been shown that non-salmonids can serve as reservoirs for the infection [200].

Other miscellaneous minor bacteria of interest not listed in the table but briefly summarized here are based on observations by expert reviewers. These include opportunistic infections by *Aeromonas hydrophila*, *Pseudomonas* sp., and *Carnobacterium maltaromaticum* (R. Holt, pers. comm.). *Vibrio anguillarum*, a serious fish pathogen of juvenile marine fishes, was associated with PSM in Chum and Coho Salmon broodstock in Washington State during transition to freshwater (B. Stewart, pers. comm.). Similar cases occurred with hatchery broodstock returns in Alaska in Coho Salmon in the 1980s and 1990s [201,202] and Chinook Salmon in 2003 [203]. Furthermore, in this state, vibriosis was the primary diagnosis for disease and PSM in wild adult Coho Salmon returns to a freshwater systems in 2019 [204] and Pink Salmon from in 2020 [205]. All the cases in Alaska were implicated in poor environmental conditions, such as high water temperatures likely accompanied with low dissolved oxygen or high fish densities.

## 4. Microparasites

**Table 3:** Review of microparasite pathogens in adult Pacific Salmon (*Oncorhynchus* spp.) in freshwater from California to Alaska. Major: significant disease or other concerns, and widespread. Minor: restricted outbreaks, although they may cause severe lesions or morbidity.

	Microparasite	Species Affected	Source (F/M)	System Affected	Disease Description	Location (State or Province)
**MAJOR**	**Ciliophora**					
	*Ichthyophthirius multifiliis* (White Spot)	All	F	I, Gi	Gill/skin hyperplasia and necrosis	worldwide
	**Ichthyosporea**					
	*Ichthyophonus* spp.	All	M, F	Sys	Asymptomatic, granulomas in organs and muscle	worldwide
	**Kinetoplastea**					
	*Cryptobia salmositica*	All	F	H, S	Anemia, splenomegaly, icterus	CA, OR, WA, BC
	**Myxozoa**					
	*Ceratonova shasta*	SO, CH, CU, CO, PI	F	Sys, G	Enteritis, extra-intestinal infections (kidney, liver, gill)	CA, OR, WA, AK, BC
	*Parvicapsula minibicornis*	SO, CH, CU, CO, PI	F	R, Gi	Glomerulonephritis, branchitis, sockeye mortality	CA, OR, WA, AK, BC
	*Tetracapsuloides bryosalmonae* (Proliferative Kidney Disease)	CH, CO, PI, SO, CU	F	R, S, C	Nephritis, splenomegaly, exophthalmia, ascites, anemia, vascular necrosis, immunocompromization	CA, OR, WA, BC, AK
	**Oomycota**					
	*Saprolegnia* spp.	All	F	I, Gi, Sys	Dermatitis, systemic, secondary infections	worldwide
**MINOR**	**Ichthyosporea**					
	*Dermocystidium* spp.	CH, SO, CO	F	Gi, I	Asymptomatic, gill hyperplasia, granulomas, respiratory distress	OR, WA, BC, AK
	*Sphaerothecum destruens* and similar parasites	CH, CO, SO, CU	F	L, R, S, C, V	Systemic granulomatous disease, organomegaly	CA, OR, WA, BC, AK
	**Microsporidia**					
	*Enterocytozoon schreckii*	CH	U	G	Enteritis	OR
	Renal microsporidium	CU	F	R	Glomerulonephritis	AK
	*Loma salmonae*	All	F, M	Gi, K, S, Re	Xenomas, branchitis	CA, OR, WA, BC, AK
	**Myxozoa**					
	*Henneguya salminicola*	All	U	M	Intramuscular cysts	OR, WA, BC, AK
	*Kudoa thyrsites*	CH, PI, CO, SH	M	M, C	Postmortem myoliquefaction	AK, BC
	*Myxidium truttae*	CO	U	L	Hepatitis	AK
	*Myxobolus* sp., *M. arcticus*, and *M. cerebralis*	CH, SO, CH	F	N, M	Skeletal deformities, neurologic abnormalities	CA, OR, ID, WA, BC, AK
	*Myxobolus squamalis*	CO, CU	F	I	Dermal cysts, secondary infections	CA, OR, ID, WA, BC, AK
	*Parvicapsula kabatai*	PI, CO	U	R	Tubulointerstitial nephritis	WA, BC

Species affected: Sockeye (SO), Chinook (CH), Chum (CU), Coho (CO), Steelhead (SH), Pink (PI). Source of infection: M = marine, F = freshwater, U = unknown. System affected: G = gastrointestinal, I = integument, R = renal, M = musculoskeletal, P = peritoneal, U = urinary, C = cardiovascular, N = neural, H = hematopoietic, S = spleen, V = visceral, Re = reproductive, Gi = gill, L = liver.

Microparasites, as defined by Brown, include small, often unicellular organisms that multiply directly within their hosts [206]. This section focuses specifically on protozoa, Myxozoa, Microsporidia, Mesomycetozoa (Ichthyosporea), and Oomycota that can cause severe disease and significant PSM in adult Pacific salmon freshwater returns. The complex life cycles of some of these microparasites, with transmission occurring in freshwater environments, marine habitats, or both, create diverse epidemiological patterns. All Pacific salmon species demonstrate varying levels of susceptibility to these infections. The physiological stress of spawning migration, coupled with the natural immunocompromised state associated with sexual maturation, creates a window of enhanced susceptibility to infections and disease manifestation. This is further exacerbated by environmental factors that continue to be problematic with climate change. The following section details the specific microparasites affecting adult spawning Pacific salmon, their geographic distribution, host range, pathological effects, and management implications (Table 3).

### 4.1. Major Microparasites

#### 4.1.1. Ciliophora

##### *Ichthyophthirius multifiliis* (Ichthyophthiriasis or White Spot)

The ciliate *Ichthyophthirius multifiliis* is an obligate parasite of a broad range of freshwater fishes throughout the world. It has caused several outbreaks and high PSM in salmon, particularly when water temperatures are elevated. It has a direct life cycle with an off host stage, in which a free-swimming theront enters the epithelium penetrating to the basal germinal layer, differentiates into a feeding trophont that increases in size (200–800 µm), exits the fish as a tomont that attaches to substrate and produces a proteinaceous cyst, and undergoes division to produce hundreds of theronts that will exit the cyst to begin the cycle again [207]. The direct life cycle is affected by temperature and can be completed in 16 to 18 h at 22–25 °C, but takes as long as four days to complete at temperatures under 13 °C. Elevated water temperature and crowding influence transmission and disease progression. Indeed, mortality associated with this pathogen is strongly linked to temperature [208], reduced flow, and water exchange rates (W. Cox, pers. comm.).

Diagnosis can be conducted by microscopic examination of wet mount preparations to observe live parasites with cilia movement and a “horseshoe” shaped macronucleus. Macroscopic clinical signs can include grossly visible white foci on exterior surfaces, skin discoloration, increased mucus production, and flashing behavior. Microscopically, affected tissues (gill and skin) display epithelial hyperplasia and necrosis. Diagnosis can also be performed using molecular methods [207,209,210]. Epithelial lesions result in respiratory and osmotic imbalances of the affected fish [211]. Bacterial infection is a common co-infection during ichthyophthiriasis. Epithelial disruption is one likely bacterial transmission factor however bacteria (including columnaris-causing bacteria) can also be associated with the parasite [212,213,214]. Cellular and humoral immunity has been documented in recovered fish leading to acquired protective immunity [215].

Epizootics of *I. multifiliis* in PNW adult salmon have been documented in numerous incidents, ranging from California [216] to Sockeye Salmon spawning channels in northern British Columbia [13]. In 2002, both *I. multifiliis* and columnaris disease were associated with the death of an estimated 33,500 adult Chinook Salmon in the lower Klamath River of northern California [144], and, in general, this parasite has been very problematic in California (W. Cox, pers. comm.). Low flows, elevated temperature, and a large returning population of salmon were contributing factors in the epizootic [143,144]. Flow is particularly critical because the infection is contracted by free swimming theronts released from off-host cysts and hence density of the theronts play a key role in severity of infections [217]. Warmer waters accelerate the life cycle and reproduction of the parasite up to the higher thermal limit of the parasite (around 28 °C).

Most treatment options used for other fishes—e.g., elevated temperatures, formalin baths, are not practical with adult salmon, leaving increasing water flow/exchange or reducing fish density as the practical options. Hence, in response to this incident in California, the U.S. Bureau of Reclamation has performed several late-summer flow tiered (primary, preventive pulse, and emergency) augmentations from the Trinity Reservoir to reduce the probability of a similar event [218]. In 2014, in-river monitoring demonstrated a high prevalence of severe ichthyophthiriasis infections in returning adults in the lower Klamath River, which prompted an emergency augmentation flow response [216]. Increased parasite DNA in 2014 river samples also corroborated adult salmon observations [209]. No fish kill or overt delayed mortality occurred in 2014. Similar to the Klamath River, high mortality, with *I. multifiliis* and *Flavobacterium columnare*, has also been documented in the California central valley’s Butte Creek in 2021. An estimated 91% of the threatened-status spring Chinook Salmon died in this epizootic [219]. A management challenge that remains for adult Pacific salmon conservation is the reduction in the infectious stage of the parasite(s) infectious dose(s) and environmental conditions conducive to the development of epizootics in returning salmon.

#### 4.1.2. Mesomycetozoa (*Ichthyosporea*)

##### *Ichthyophonus* spp.

*Ichthyophonus* spp. (ichthyophoniasis) are obligate parasites that have undergone several taxonomic revisions and are currently classified as mesomycetozoan parasites at the animal-fungal boundary [220]. The type species *Ichthyophonus hoferi* [221] was inadequately described and the type material no longer exists. Species have been described based on morphology, but this can be highly plastic and isolates have been reported from a wide range of some 140 or more host species, which has led others to conclude that current reports of the genus *Ichthyophonus* represent a cryptic species complex [222,223,224,225]. Hence, we refer to the parasite only by the generic name, *Ichthyophonus.* The parasite is mostly of marine origin, but freshwater isolates exist [17]. Piscivorous fishes primarily become infected via ingestion of infected prey, but co-habitation studies have also shown horizontal transmission in rainbow trout [17]. Additionally, the life cycle for infecting planktivorous fish is unknown, and we currently do not know the source of infections for Yukon Chinook Salmon in Bering Sea [15].

Reports of infections in salmonids have included all five Pacific salmon species [17,226,227,228,229]. However, studies have shown that Chinook Salmon from the Yukon River are more susceptible to the disease and exhibit higher mortality than all other salmonid stocks, resulting in chronic infections and high mortality [230,231]. Infections in adult freshwater Yukon Chinook Salmon returns result in disseminated disease and significant PSM [15,16,232,233] that has been estimated as high as 60% [15]. In Alaska, this is the most significant fish disease associated with population declines. Over 63% of diagnostic cases involving adult Chinook Salmon statewide since the 1980s have involved ichthyphoniasis and/or PSM of Bering Sea stocks (mostly Yukon), which is drastically higher than any other disease or PSM events for any Pacific salmon species adult returns in this state (J. Ferguson, pers. comm.). *Ichthyophonus* was first detected in Alaska from a Yukon River Chinook Salmon in 1988 [234], followed by multiple years of subsistence reports from the middle Yukon River that affected Chinook Salmon in their harvest smelled “fruity”, did not dry properly when smoked, and had white foci in the flesh, heart, and other organs. The disease has become a common episodic problem with severe impacts on subsistence harvesters and fish populations. The first major epizootic occurred in the early-mid 2000s [15], which has recently resurged at unprecedented levels [235]. Ichthyophoniasis has been associated with high en route mortality of adult Yukon River Chinook Salmon since 2020 [235], where there has been substantial differences between sonar counts of adult Yukon Chinook Salmon returns at the mouth of the river compared to the Canadian border passage that was most parsimoniously explained by PSM [235]. The other sources of loss being fish harvest or straying were deemed unlikely, because fisheries were closed and telemetry studies have shown minimal straying. Indeed, fishery managers and researchers have estimated this recent en route PSM to be as high as 43–53% equating to about 34,000–35,000 fish annually over a several year period [236,237,238]. This has resulted in significant hardships for the subsistence fishers who rely on these iconic fish for food and cultural identity. Ichthyophoniasis is more severe in warmer water temperatures, so these epizootics have been linked to climate change [15,16,239]. Alaska and other sub-Arctic/Arctic nations are on the forefront of climate change with the poles warming 2–4 times faster than the global average, a phenomenon known as polar or Arctic amplification [240].

Disease can range from subclinical to chronic, severe granulomatous inflammation, necrosis, and fibrosis that can be obliterative, or it can even be acute with direct mortality [241]. Severity depends on general fish health and stress where infections are typically chronic and persistent. Fatal disseminated disease occurs in susceptible hosts, such as Yukon River Chinook Salmon. Typically, there is a strong inflammatory response to the parasite, which usually results in grossly visible granulomas or granulomatous inflammation as the host attempts to encapsulate the schizonts (resting spores). Granulomas are comprised of a wall of collagenous stroma and fibroblasts containing variable numbers of lymphocytes, epithelioid macrophages, eosinophils, and neutrophils [242,243]. Lesions can appear white, yellow, or brown in blood filtering organs, especially the heart, and skeletal muscle. Disseminated infections in diseased Yukon Chinook Salmon results in parasites in every organ system, including the brain. Performance changes seen in subclinical fish included include reduced swimming performance [244], increased energetic costs [245], and reduced aerobic fitness scope.

Ichthyophoniasis can be presumptively diagnosed by grossly observing characteristic white lesions in blood rich organs, with a predilection for heart, which can result in severe cardiomegaly. However, differential diagnoses include BKD, PKD, nephrocalcinosis, mycobacteriosis, and larval cestodiasis and trematodiasis. Historically the reference standard for diagnosis has been identification of the parasite in culture of the heart, where the parasite is visualized with an inverted microscope to view resting stages called various names, such as macrospores or schizonts ranging from 20–200 µm in diameter and germinating hyphae (germination tubes). Additionally, diagnosis can also be based on histology, with the characteristic fungal-like structures in various tissues, including the heart, kidney, and skeletal muscle that stain periodic acid-Schiff- (PAS) positive, which is a not a specific stain, but the parasite can be confirmed by in situ hybridization in tissue sections [246]. More recently, PCR and qPCR tests have been used as a screening method [247,248] that can also be used as a confirmatory test. However, the qPCR assay is over 100 times more sensitive and has been semi-validated following World Organisation for Animal Health (WOAH) guidelines and can be used as a proxy to quantitate infections [248]. There are currently no treatments for controlling the disease and mortality of fish due to this parasite. However, Alaska is currently developing an in-season monitoring program with a risk assessment model to better inform managers on modifying strategies to mitigate the impact on the population in fisheries.

#### 4.1.3. Kinetoplastea

##### *Cryptobia salmositica* 

Woo provides a thorough review of *C. salmositica* infections in salmonid fishes [249], with several references by Bower, Beck and Margolis cited therein pertaining to *Oncorhynchus* species in the PNW. Similar to certain *Trypanosoma* species in mammals, the infection presents with massive numbers of hemoflagellates in the blood. The parasite infects all *Oncorhynchus* species in the PNW, as well as some non-salmonid fishes; sculpin are considered important reservoir hosts. Infections occur in Pacific salmon from northern California to British Columbia. It is transmitted by a freshwater leech (*Piscicola salmositica*) but can be transmitted directly when fish are held in captivity. The latter mode of transmission includes brief cohabitation when netting fish together [250]. Reports of associated PSM range throughout the PNW, particularly in Chinook Salmon, whereas some populations appear to tolerate very heavy infections. Infections and morbidity also occur in juvenile salmonids in freshwater and may occur after fish are later transferred to seawater net pens. One of us (N.H.) has observed outbreaks in adult Chinook Salmon in Washington State. Consistent with other reports, infected fish are anemic and have severely enlarged spleens. The parasite causes anemia through hemolysis, and the skin of infected fish may appear yellow-gold appearance (jaundice). The BCFHD lists five cases linked to PSM from five locations in British Columbia, including Sockeye, Pink, and Coho Salmon. In Chinook Salmon in Oregon, *C. salmositica* was the primary pathogen seen in a PSM occurrence in 2004, with BCWD, saprolegniasis, and IHNV co-infections (C. Banner, ODF&W, pers. comm.).

Given the intensity of infections, simple blood smears or wet mounts are effective and practical diagnostic methods for clinical cases, where large numbers of flagellates are observed in infected fish. Woo reviews potential control measures [249], but application of these with adult fish held in captivity would be difficult. Returning adult Chinook Salmon injected under a United States Food and Drug Administration (USDA) exemption with isometamidium chloride, an anti-trypanosomal drug, experienced decreased PSM [251]. The parasite survives poorly at 20 °C, but this treatment would not be practical with adult salmon. Given the ease of direct transmission, once identified, infected fish should be segregated from uninfected populations.

#### 4.1.4. Myxozoa

The phylum Myxozoa contains several species that are pathogenic to salmonids. Three species, *Ceratonova shasta*, *Parvicapsula minibicornis*, and *Tetracapsuloides bryosalmonae*, are designated here as Major pathogens. In addition, other species will be reviewed that we designate as Minor pathogens, including *Myxobolus* spp., *Parvicapsula kabatai*, *Kudoa thyrsites*, *Myxidium truttae*, *Henneguya salminicola*, and *Chloromyxum* sp.

Almost all myxozoans have complex life cycles involving both fish and invertebrate hosts. Infections by *H. salminicola* and the *Myxobolus* species in our review very likely occurred in juveniles in freshwater and the infections persisted to adulthood [252,253], while others may be acquired in the marine environment (*K. thyrsites*) or after return to freshwater (e.g., *C. shasta*).

##### *Ceratonova shasta* 

*Ceratonova* (syn. *Ceratomyxa*, Noble 1950) *shasta* infects freshwater salmonid fishes and is endemic to anadromous fish in tributaries of the PNW, including Alaska [254]. Infection by *C. shasta* often results in necrotic enteritis and anemia in juvenile salmon. Extra-intestinal disease with granulomas, abscesses (boils), and necrosis, can also occur. Other clinical signs can include body darkening, swollen hemorrhagic vents, abdominal distention from ascites. This disease is a significant mortality factor for juvenile Chinook Salmon in the Klamath and Feather River [255,256,257,258]. It has also been reported in Coho, Sockeye, and Chum Salmon. *Ceratonova shasta* has a complex life cycle, involving an invertebrate polychaete host (*Manayunkia occidentalis*) [259,260]. Infected polychaetes release actinospores into the water where they attach to the salmon’s gill epithelium, invade into the blood, replicate, and later migrate to the intestinal tract for further multiplication and sporogony [261]. Depending on parasite genotype and density, innate host resistance, and water temperature, infected fish can develop varying degrees of enteritis and associated anemia [262,263,264,265]. Based on studies of juvenile fish, after approximately two weeks post-infection, the parasite can form the myxospore stage that is typically released after the fish dies or in feces from the intestine. If ingested by the filter-feeding polychaete it completes the life cycle after invading the worm’s gut epithelium [266]. In the Klamath River, *M. occidentalis* inhabits tubes constructed of fine sediment and mucus in a variety of lotic conditions with highest densities found in low velocity areas [267]. *M. occidentalis* is reported to have an annual reproduction cycle [268]. Adult salmon with the myxospore stage, often released after death, are the likely source of the annual polychaete infection [269,270].

Despite the high prevalence of infection in adult Chinook Salmon, the parasite is generally not associated with PSM, but there are exceptions. This includes the Klamath and Sacramento River systems in California [255,269,271]. Other pathogens tend to be the drivers in PSM in these rivers. It is possible that declining water temperatures in the fall and winter reduce the degree of enteritis and extraintestinal spread and slow sporulation [265]. Likewise, *C. shasta* is very prevalent in adult Chinook Salmon in the Willamette River basin in Oregon [10,105], where most infections are confined to the intestine or stomach and many infections were represented by only presporogonic forms [270]. This predominance of presporoganic infections without detectable spores for many months up to spawning contrasts with infections in juvenile salmonids, where the parasite sporulates within weeks after infection. Kent et al. showed that the parasite actually sporulates after death in post-spawned fish [270]. A similar situation was documented in salmon in California at the population level, where decomposed carcasses had a much higher prevalence of myxospores than newly spawned salmon [269].

Review of our archives of histology for Chinook Salmon in the Willamette River system showed that only a few fish had extraintestinal infections; about 2% in the liver or kidney and only one fish had gill infections. Prevalence of the infection actually increases in adults leading up to spawning [9,10]. Indeed, using odds ratio analyses, Benda et al. reported that a spawned Chinook Salmon from Oregon were seven-times more likely to have *C. shasta* than PSM fish [9].

**Figure 4:**
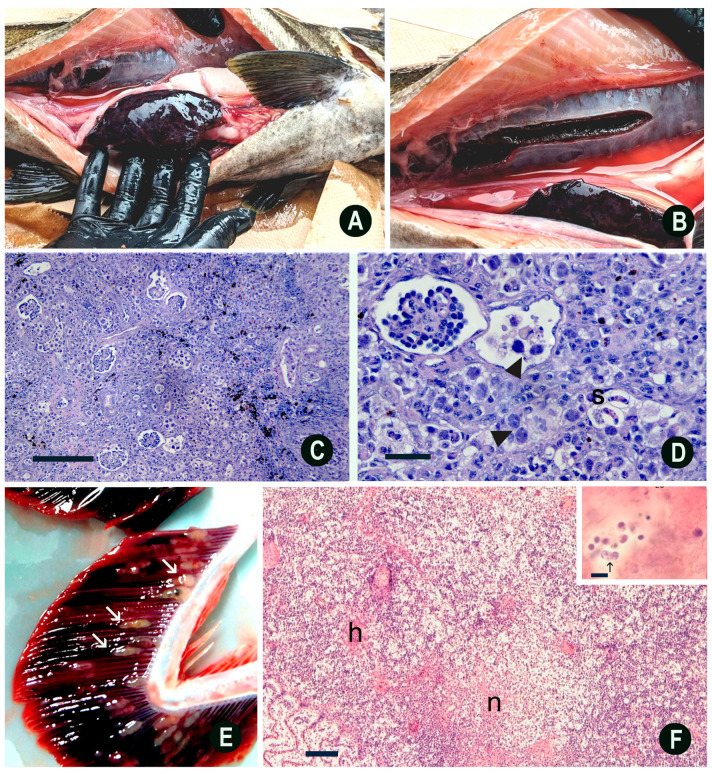
Extraintestinal infections of *Ceratonova shasta*, gross and microscopic images of kidney in adult Chinook Salmon and gill in Sockeye Salmon. (**A**,**B**) Severe renosplenomegaly with a mottled appearance in a post-spawn fish. (**C**) Kidney. Severe, widespread tubular loss with intraluminal cellular casts and marked interstitial granulomatous inflammation. Remaining tubules are at varying stages of degeneration, attenuation, and regeneration. (**D**) Within the lesion, there are frequent parasites including presporgonic stages (arrowheads) and two spores (S). H&E. (**C**), bar = 100 µm. (**D**), bar = 10 µm. (**E**) Gill section of Sockeye Salmon with dozens of raised, pale tan to yellow lesions within the gill filaments (arrows). (**F**) Gill histology of Sockeye Salmon with prominent *C. shasta* infection effacing the architecture of the primary filaments with multifocal foci of hemorrhage (h) and gill necrosis with numerous parasites (n). Bar = 100 µm. Insert at higher magnification shows a spore (arrow) and presporogonic stages. H&E.

We hypothesize that the lack of significant PSM in these fish may be because only a small percentage developed extraintestinal infections. In contrast to the Willamette System and California, adult Chinook Salmon from one hatchery on the Deschutes River in Oregon have consistently shown severe systemic lesions in association with high PSM. Grossly, these fish have enlarged spleens and kidneys with mottled pallor (Figure 4A,B). The internal macroscopic changes are suggestive of BKD (Figure 4C,D), but these same fish consistently tested low for *R. salmoninarum* by ELISA by the ODF&W. In British Columbia, the BCFHD lists six cases of PSM in adult Chinook Salmon in rivers with *C. shasta* listed as the etiologic agent. Here the diagnosticians recorded macroscopic liver lesions and spores, consistent with our hypothesis that extra-intestinal infections of this parasite are linked to PSM.

Another unusual presentation associated with PSM occurred in Alaska, where numerous multifocal nodular lesions were present in the gills of wild Sockeye Salmon with severe lethargy, unresponsiveness, and signs of respiratory distress, and mortality (Figure 4E) [272]. This was a heavy infection with severe, diffuse, necrotizing and fibrotic branchitis and expansion of the gill filaments (Figure 4F). Fish were also co-infected with low levels of *Loma salmonae* and *Ichthyophonus* that may have been contributory to the primary diagnosis of branchial ceratomyxosis. PCR and deoxyribonucleic acid (DNA) sequencing conducted by one of us (J. F.) confirmed that the myxozoan from branchial nodules were indeed *C. shasta*.

##### *Parvicapsula minibicornis* 

*Parvicapsula minibicornis* was first described from adult Sockeye Salmon in the Fraser River system, British Columbia [273]. Infections were associated with kidney lesions and the high prevalence raised concerns about a possible association with elevated PSM. Subsequent studies revealed the occurrence of the parasite in adult salmon originating from various larger rivers draining into the Pacific Ocean from California to British Columbia [14,274,275]. More recently, *P. minibicornis* co-infection was associated with a few wild adult Yukon River Chum Salmon with clinical PKD [276].

In the kidney of affected Sockeye Salmon, glomerular capillaries are distended and occluded by large numbers of parasitic trophozoites. In fully developed infections, large numbers of mature myxospores occur in the lumen of the renal tubules. Concurrent thickening of the glomerular basement membrane may result from persistent antigenic stimulation and immune complex deposition. Focal to segmental coagulative necrosis of the epithelial lining with occasional proteinaceous casts occurs sporadically in renal tubules [277,278]. The infection, confirmed by using ISH, also occurs in Sockeye Salmon gill and is associated with a multifocal to diffuse branchitis [279]. In Chinook Salmon in the Willamette River, similar to Sockeye Salmon, the infection was associated with a severe glomerulonephritis [11].

There is no evidence of infection in adult Sockeye Salmon prior to migration into the Fraser River and the infection is first detected in adult salmon shortly after migration into the river, indicating exposure to the parasite occurs in the lower river or estuary. Subsequently, the severity of infection increases with distance from the river mouth or with time in the river [14,279,280]. Physiological changes associated with parasite-induced kidney damage in spawning Sockeye Salmon include a negative correlation between plasma osmolality and the number of parasites in renal tubules. A positive correlation between plasma lactate and the severity of gill pathologic changes is also reported [279]. The severity of infection in Fraser River Sockeye Salmon was proportional to the accumulation of thermal units (degree-days) following a controlled exposure to the parasite, and the swimming performance of heavily infected adult Sockeye Salmon was significantly reduced compared with uninfected controls indicating a decreased ability to recover from exercise [281]. In the Willamette River in Oregon, spawned Chinook Salmon held in cool water tanks were less likely to be infected with *P. minibicornis* than those spawned out-planted fish [9], although it is not clear whether the experimental manipulations affected exposure to the parasite. In Fraser River Sockeye Salmon, infection with *P. minibicornis* may be an important factor contributing to PSM, particularly when combined with elevated river water temperatures and prolonged river residence time such as brought on by premature migration of the salmon into the river [279,282]. The relationship between temperature and infection severity suggests negative consequences that may be expected with rising river temperatures under climate change.

In addition to Sockeye and Chinook Salmon, the parasite has been reported from Steelhead Trout and Coho and Pink Salmon from the Fraser River, Columbia River, Klamath River, and Sacramento River drainage basins and some smaller drainages in western North America [274,275,283]. The infection was detected by PCR in adult Sockeye and Coho Salmon from the Columbia River but unlike in the Fraser River, the parasite was not detected in histological material from these fish [275]. It was also detected by DNA sequencing of adult Chum Salmon with clinical PKD from the Yukon River in Alaska [276]. There is evidence of genetic heterogeneity among isolates of the parasites obtained from diverse river drainages and host species [274], although the significance of this trait with respect to differences in pathogenicity is not known. Much of the research effort in the Klamath and Sacramento River basins focuses on infections in out-migrating juvenile salmon [283].

Occurrence of the parasite among several river systems is likely a reflection of the distribution of its invertebrate host, *M. occidentalis*, [259,284], the same polychaete host for *C. shasta*. Although this species has only been reported from the Klamath and Willamette Rivers, the fish infection data indicates the worm occurs over a much broader range among river systems between California and British Columbia, and possibly into Alaska. The coincident role of *M. occidentalis* as the invertebrate host of *C. shasta* [260], explains the broad epizootiological similarities of *P. minibicornis* and *C. shasta* infections among anadromous salmonids in western North America.

In addition to histology, the infection is diagnosed through the use of specific qPCR tests [285]. Conventional PCR using species-specific primers with or without sequencing of the PCR product may also be used [274,280]. Microscopic examination of stained tissue sections, particularly the kidney, is used to assess the infection severity and associated glomerular lesions.

##### *Tetracapsuloides bryosalmonae* (Proliferative Kidney Disease)

*Tetracapsuloides bryosalmonae*, formally “PKX organism”, is a malacosporean myxozoan parasite that causes proliferative kidney disease (PKD), which is an important disease in salmonids [286,287]. PKD has been reported in both wild and captive salmonids and several other species including Mountain Whitefish (*Prosopium williamsoni*) and Northern Pike (*Esox lucius*) in the PNW and Newfoundland; Trout, Atlantic Salmon and European Grayling (*Thymallus thymallus*) in Europe, including Finland and Sweden; and Arctic Char in Iceland. Incidence and severity of PKD is linked to increased temperatures where values above 15–16 °C have been shown to enhance both parasite replication and disease pathogenesis in the fish host [288,289,290,291]. Therefore, this disease may be expanding and emerging in new areas due to climate change [292,293,294].

This parasite utilizes bryozoans as definitive hosts, instead of the oligochaete worms typically used by myxosporeans [295,296]. Waterborne spores are released from bryozoans, usually *Fredericella* spp. and *Plumatella* spp. [297,298]. These spores are dispersed with either passive or active mechanisms [299,300] and infect an intermediate fish host primarily through the gills [301]. The parasite migrates through the vasculature with a predilection for the posterior kidney, where it ultimately migrates to the tubules and establishes a persistent coelozoic sporogonic phase in the lumen of renal tubules and tolerant hosts excrete mature malacospores into the environment via urine to infect bryozoans and complete the life cycle [287,298,302].

Temperature increase induces transition from covert into overt infection where infectious stages of *T. bryosalmonae* develop and are released into the water by bryozoans. Variable mortality (5–90%) occurs at elevated water temperatures, usually >15 °C, and co-infections often exacerbate mortality. Many fish will recover in the absence of these co-infections, including those with macroscopic renal lesions and moderate anemia.

Typical gross clinical signs of disease include pale gills, a uniformly swollen kidney and/or spleen that may be gray or mottled, exophthalmia, ascites and anemia. Cytologically, the parasite consists of amoeboid PKX cells (10–20 µm) with foamy cytoplasm, distinctive cell membrane and 1 mother cell (primary) nucleus with 1–7 daughter cells [303,304]. The histopathology typically involves proliferative and granulomatous nephritis, vascular necrosis and thrombi with eosinophilic PKX cells among the kidney interstitial cells that are often surrounded by attached host macrophages. For intolerant or partially intolerant salmonids, blood-migrating stages can invade the renal interstitium and trigger an aberrant histozoic extrasporogonic proliferation, which causes a chronic immunopathology leading to host immunocompromisation, and hence fish are often predisposed to secondary infections [305,306,307]. Surviving fish can develop protective immunity and may clear the infection and heal damaged tissues [308,309,310,311].

In addition to various locations in California and Oregon, the parasite is enzootic in streams on eastern Vancouver Island where it infects and causes disease in juvenile Chinook and Coho Salmon. Regarding adult salmon, in the Quinsam River on Vancouver Island, the prevalence in spawning Pink Salmon, when measured by using PCR, ranged from 43% to 100% between 2003 and 2008. Although PKD (i.e., renomegaly) was not detected, heavy infections were observed in kidney, spleen and gill by using in situ hybridization [312,313,314]. The monthly prevalence (by PCR) in spawning Pink Salmon from the Quinsam River in 2023 increased from 0% in August to 100% in October (S. Jones, unpublished data). In the same year, the prevalence in spawning Chinook Salmon and coho salmon collected from the Quinsam River in October and November ranged from 53% to 100% (S. Jones, unpublished data). These data support the use of broodstock screening to confirm the presence of *T. bryosalmonae* in a watershed, and therefore the potential risk of PKD to juvenile salmonids in that watershed. In Alaska, the parasite has been reported to cause clinical disease in five wild adult Yukon River Chum Salmon returns, where this was confirmed in two of the submitted fish that also had a co-infection with *P. minibicornis* [276].

Diagnosis is based on clinical signs and the detection of the parasite in cytology or histology [268]. Parasite DNA can also be detected in all organs by PCR and PKX cells can be observed in kidney, spleen and liver of infected fish by immunohistochemistry [304]. Differential diagnoses to consider include BKD and nephrocalcinosis. Juvenile salmonids have been treated successfully with oral administration of fumagillin [313,315], but this would not be feasible with adult salmon, both in the wild and hatcheries.

#### 4.1.5. Oomycota

##### *Saprolegnia* spp.

Oomycetes of the genus *Saprolegnia* commonly infect returning adult salmon and can be major contributors to PSM, particularly for fish with traumatic lesions from their upriver migration and spawning behavior. Co-infections with more lethal pathogens are common because saprolegniasis causes large disruptions to the skin barrier. *Saprolegnia* spp. infects all *Oncorhynchus* species throughout the North Pacific, with the most common species being *S. parasitica* [316].

Oomycetes, commonly referred to as water molds, are eukaryotes that are part of a large taxonomic group that includes certain algae and flagellates [317]. While saprolegniasis is often called a “fungal disease” due to previous classification, oomycetes are now known to not be closely related to fungi. Non-septate mycelia growing on fish tissues develops club-shaped sporangia, structures at the tip of the mycelia used for asexual reproduction that are full of infectious zoospores. Both mycelia and zoosporangia are easily visualized using a compound microscope. The classic presentation for saprolegniasis in adult Pacific salmon is dermatitis with large, circular lesions with fuzzy, white to brown cotton-like growths on the body, particularly in areas of the head and fin bases. Systemic disease can also occur with mycelial masses that invade the gut and viscera causing peritonitis, hemorrhage, necrosis and adhesions. It also commonly grows in traumatic injuries from upriver migration, including seal and lamprey bites, ulcers and abrasions from rocks and barriers, and fin and skin avulsions and punctures from human fishing activities. Saprolegniasis is chronic and can persist for months, with lesions often slowly growing and coalescing. Mycelia invade the dermis and muscle tissues as the infection progresses. Co-infections with bacterial pathogens including *A. salmonicida* and columnaris-causing bacteria are common, and these bacteria are likely to infect fish through the large areas of skin disrupted by saprolegniasis. Fish may die from secondary disease before saprolegniasis, as many diseases progress significantly faster than this specific disease. In severe cases, histologic findings include a hyperplastic to ulcerated epidermis overlayed by a thick hyphal mat, which extends into the deeper tissues, like skeletal muscle, as the lesion progresses, resulting in tissue necrosis and edema [318,319]. Histochemical stains like Grocott–Gömöri’s methenamine silver (GMS) or PAS can be useful in visualizing the hyphae in histologic sections.

Oomycetes are ubiquitous in aquatic environments and do not require a living host to grow. Pacific salmon carcasses at spawning grounds are often entirely covered in these water molds, as can PSMs in hatchery holding containers. These carcasses are a source of high *Saprolegnia* spp. exposure as adult salmon return to their natal streams or are held in hatcheries. Host immune status is the critical factor in infection progression and disease development. Returning adult salmon are undergoing senescence and are immunocompromised, predisposing them to infections that healthy salmon do not typically succumb to [11,12,320]. Combined with a high rate of physical injuries from upriver migration, the slow wound healing and reduced immune status caused by senescence make adult Pacific salmon highly susceptible to saprolegniasis. It is rare for diagnosis of saprolegniasis to progress beyond observation of gross lesions, although sometimes wet mount visualization of mycelia and sporangia are used to confirm, which may be needed for the systemic version of the disease. *S. parasitica* can be cultured with common mycology media, including potato dextrose agar. *Saprolegnia* spp. can be confirmed with molecular diagnostic testing including qPCR and sequencing [321,322].

Historically formalin and malachite green in combination as an immersion treatment were effective in controlling saprolegniasis in Pacific salmon broodstock; however, malachite green is no longer used due to human safety concerns [316]. Formalin treatment alone will not treat saprolegniasis, but it is effective in reducing injuries from developing saprolegniasis as well as preventing progression of infection. A common regimen for treating adult Pacific salmon broodstock held in a hatchery prior to holding is 167 part per million (ppm) formalin for 1 h, and subsequent treatment with frequency varying from weekly to daily depending upon observation of the fish. If oomycetes are observed, the frequency should be increased. Adult Pacific salmon generally tolerate daily formalin treatments at 167 ppm for 1 h without adverse effects at low temperatures. Effective treatment frequency is evident when fish with wounds do not develop secondary saprolegniasis. In the experience of many fish health professionals over the years, formalin immersion is observed to be significantly more effective at controlling saprolegniasis than hydrogen peroxide immersion. In Oregon, even with the effectiveness of formalin, many hatcheries halted its use due to disposal requirements of formalin-treated water before entering streams (R. Holt, pers. comm.). However, 35% hydrogen peroxide is extremely corrosive and represents a significant safety concern. Non-pharmaceutical methods of controlling saprolegniasis in hatchery settings include selection of broodstock without injuries that will be prone to secondary saprolegniasis, prompt removal of mortalities in hatchery settings, and 3% salt solution immersion for 1–3 min. Increased water flows and cleaning of units can also help.

As with other infectious agents, several knowledge gaps remain regarding *Saprolegnia* spp. in adult spawning Pacific salmon. This may include: species and strain differences of oomycetes in the different species of Pacific salmon between different geographical locations and holding conditions; alternative treatments to formalin, which is vulnerable to increased regulation and subsequent decreased availability because of environmental concerns about chemical discharges in water; and the dynamics of co-infections between *Saprolegnia* spp. and other pathogens, particularly the degree to which saprolegniasis predisposes fish to bacterial, viral, and parasitic co-infections.

### 4.2. Minor Microparasites

#### 4.2.1. Mesomycetozoa (*Ichthyosporea*)

##### *Dermocystidium salmonis* 

Several members of the genus *Dermocystidium* infect fishes. This genus is a member of the Class Mesomycetozoa (previously *Ichthyosporea*), along with two other genera *Ichthyophonus* and *Sphaerothecum*, which infect fishes. *Dermocystidium salmonis* has been reported infecting the gills several salmonid fishes. Regarding the focus of our study, a few reports of mortality associated with this protist have been reported in adult Chinook Salmon from British Columbia [323], Washington [324] and Oregon [325]. In British Columbia, according to the BCFHD, 11 cases associated with PSM were seen in Sockeye, Coho, and Chinook Salmon. One case of a *Dermocystidium*-like organism was associated with granulomas in an adult Sockeye Salmon in 1989 in Alaska [326]. The infection is common in certain rivers in adult Chinook Salmon, but there is usually little associated mortality—i.e., the less common situation with significant PSM has linked to heavy infections, low water flow and higher summer water temperatures. *D. salmonis* also may impair the ability of smolts to adapt to seawater [325].

Regarding histopathology, *D. salmonis* is an example of a relatively well tolerated parasitic infection, with minimal tissue reaction considering the location and size of the parasite. Only exceptionally heavy infections, resulting in significant damage to the gills and space occupying loss of respiratory capabilities, have been associated with significant mortality. Macroscopically, multiple white, sometimes coalescing spheres (pseudocysts) are observed in the gills. Histology demonstrates parasite aggregates between lamellae and localized inflammation and epithelial hyperplasia [325]. A pathognomonic characteristic is that individual organisms within the pseudocysts are spherical and most have a large, distinctive vacuole. Infections are mitigated by providing higher flow water with cooler temperatures in attempt to keep fish alive long enough to spawn. We are not aware of therapeutants being used with adult salmon with this infection.

##### *Sphaerothecum destruens* 

*Sphaerothecum destruens* is a debilitating infectious and systemic disease of seawater pen-reared Chinook Salmon and was shown by inoculation of naïve fish to be caused by an enigmatic organism referred to as the rosette agent [327,328]. Subsequent applications of molecular tools showed that the rosette agent belonged to a clade of organisms, including *Dermocystidium*, *Ichthyophonus*, and *Psorospermium* [329,330,331,332], later described as the class Mesomycetozoea [220]. The rosette agent was classified as *S. destruens* [333] and is recognized as a risk to fish conservation globally [334].

In Alaska, the rosette agent was diagnosed from an adult Yukon River Chum Salmon in 1999 associated with granulomatous dermatitis [326] and an adult anadromous Dolly Varden (*Salvelinus malma*) return from Nome, Alaska in 2020 that infected the renal parenchyma and was a co-morbidity among multicentric ichthyophoniasis and apparent resolving PKD [335]. The rosette agent in the latter case was confirmed by PCR. Clinical signs of the disease in Chinook Salmon held as captive broodstock in California were variable according to infection severity, and the prevalence of infection varied interannually between 0.7% and 40.1% with variable mortality among captive broodstock [336]. However, *S. destruens* has been documented in California salmonids in other situations but not associated with PSM (W. Cox, pers. comm.).

Grossly, nodules in the liver, kidney, spleen, heart and peritoneal mesentery may occur together with enlargement and pallor of the liver, kidney and spleen [334,336]. Microscopically, the infection presents either as a systematic infection with limited host inflammatory response, or as granulomatous lesions in kidney, liver and spleen. The organism occurs intra- or extracellularly in aggregates, or rosettes, within affected organs. In kidney, infections are associated with necrosis and loss of tubules, glomerular nephritis and interstitial nephritis.

**Figure 5:**
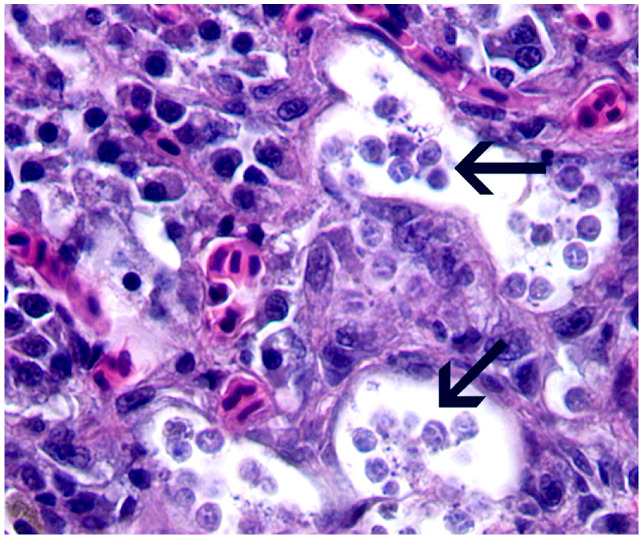
Liver of an adult Chinook Salmon from Oregon. Hepatic necrosis with dozens of spherical, multicellular protists suggestive of *Sphaerothecum* sp. (arrows) with lymphoplasmatic inflammation. H&E.

The organism is cultured in CHSE-214 cells and cultured stages are infective [327,336]. Chinook and Coho Salmon and Rainbow and Brown Trout (*Salmo trutta*) are all susceptible to experimental infections with some evidence of variations in susceptibility among the species [336]. Infections are detected by using specific PCR assays [337]. Whereas not confirmed with sequencing, multicellular spherical parasites consistent with the rosette agent were seen by histology in the livers in a few adult Chinook Salmon from the Willamette River system (Figure 5) and an adult Sockeye Salmon from British Columbia.

#### 4.2.2. Microsporidia

##### *Enterocytozoon schreckii* 

*Enterocytozoon schreckii* is a newly described microsporidian parasite that infects the intestinal epithelium of spawning Chinook Salmon, only documented thus far in Oregon [10,338]. The microsporidium has only been identified in adult Chinook Salmon with adult salmon enteritis (ASE), a condition characterized by intestinal inflammation, epithelial dysplasia, and necrosis in spawning adults [320]. *Enterocytozoon bieneusi*, a common opportunistic infection in acquired immunodeficiency syndrome (AIDS) patients shares many similarities to *E. schreckii*—e.g., infecting enterocytes of immune compromised hosts, similar morphology and taxonomic relationships based on small-subunit (SSU) ribosomal DNA sequence [338].

Histopathologic examination for developmental stages within intestinal tissues, especially pyloric ceca and middle intestine, is the primary diagnostic method. The parasite produces small (2–2.5 μm length), basophilic spherical meronts in enterocytes that develop into focal clusters of amphophilic, refractile mature spores within the cytoplasm of intestinal epithelial cells, causing cellular damage and disruption of normal intestinal architecture [10,338]. Fecal smears with FungiFluor detect mature spores and electron microscopy of tissues have also been successfully employed [10,320,338].

While observed only in adult spawning Chinook Salmon from rivers, a recent laboratory demonstrated successful transmission to juvenile Chinook Salmon through oral gavage, with transmission efficiency enhanced by host immunocompromisation. This provided evidence supporting *E. schreckii*’s potential role in ASE pathogenesis, either as a primary pathogen or as an opportunistic agent [320]. Based on these studies, it could be theorized that the parasite is transmitted via the fecal-oral route, with environmentally resistant spores released in feces and subsequently ingested by new hosts. Whereas *E. bieneusi* is recognized as an opportunistic infection in AIDS patients, the role of *E. schreckii* in ASE in salmon is not resolved, particularly because at this time the parasite has only been seen in populations with ASE and it also was observed in juvenile fish with ASE from a laboratory transmission study [320]. Other significant knowledge gaps remain regarding the complete host range, geographic distribution, and life cycle of *E. schreckii*, as it has thus far only been documented in Chinook Salmon in Oregon [10,338]. The clinical significance and pathogenesis are still being determined, particularly regarding its role in intestinal pathology and PSM [10]. The absence of other known enteric microsporidia in these fish populations simplifies differential diagnosis [338], but given the small size of the parasite and lesion paucity, a PCR test would provide a sensitive tool to better capture the epidemiology, pathogenesis, and potential control measures for *E. schreckii*.

##### Renal Microsporidium

**Figure 6:**
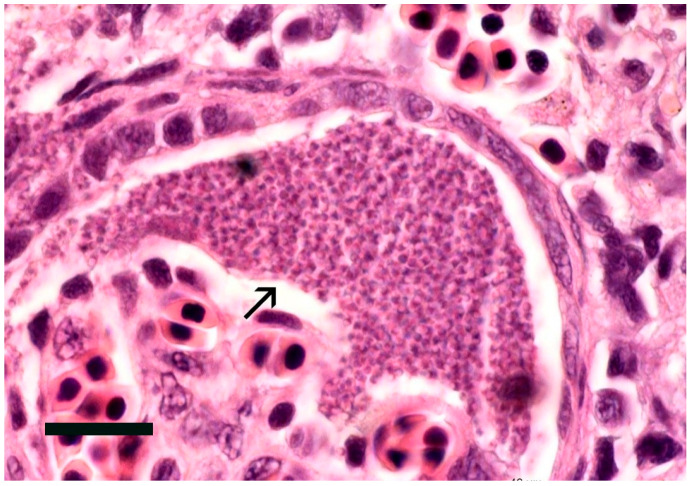
Renal microsporidium from adult Chum Salmon in Alaska, expanding Bowman’s space is a large aggregate of spores (arrow) suggestive of a xenoma with reactive (hypertrophic) parietal cells. H&E. Bar = 20 µm.

An incidental finding of a novel microsporidium was noted in 30% of Yukon River fall Chum Salmon in 2022 [339], infecting the glomerulus with no associated pathologic changes besides a space-occupying lesion (Figure 6). The organism’s SSU rDNA was sequenced and was most similar to *Ichthyosporidium weissi*. However, it was only 91%, and thus likely represents an undescribed species given the low sequence similarity of this gene based on current data in the National Center for Biotechnology Information (NCBI) database (J. F., pers. comm.; NCBI Acc. No. PV990048). Moreover, the parasite does not show the distinctive morphology of this genus, which is characterized by very large xenomas and highly pleomorphic spores. Interestingly, Burek & Underwood detected what appears to be the same parasite in fall Chum Salmon from this river system at a similar prevalence decades earlier [226].

##### *Loma salmonae* 

*Loma salmonae* is a common microsporidium in juvenile *Oncorhynchus* species in freshwater throughout the PNW [340]. Speare and Lovy reviewed this parasite and associated pathologic changes [341]. Heavy infections can cause morbidity, of note are severe infections with extensive tissue reaction in seawater net pen-reared Chinook and Coho Salmon. PSM has been documented in Sockeye Salmon in the Babine system in northern British Columbia. The primary cause was likely gill and skin infections by *Ichthyophthirius multifiliis* [13], but we have noted heavy co-infections in these affected populations (M. J. Higgins, Fisheries and Oceans Canada, Pacific Biological Station (DFO-PBS), pers. comm.). Shaw et al. surveyed adult salmon in British Columbia for the parasite, and found infections in the gills of Chinook, Coho, Chum and Sockeye Salmon [340]. Histological data from British Columbia showed that prevalence in adult Pink Salmon collected monthly between August and October increased from 7% to 77%, suggesting transmission occurred during holding in freshwater prior to spawning (S. Jones, unpublished). Review of the BCFHD revealed PSM is associated with *L. salmonae* in four cases, including Chinook, Sockeye and Pink Salmon.

In Alaska, heavy *L. salmonae* infection was one of the primary causes diagnosed for two high PSM events in two different hatchery broodstocks of Pink Salmon held for ripening in separate years and locations of the state [113,342]. However, these cases were also associated with co-infections and environmental stressors that likely pre-disposed these fish to more severe disease and mortality. Co-morbidities in the earlier case involved mixed opportunistic motile *Aeromonas* septicemia, saprolegniasis, and hypoxia due to low dissolved oxygen and high-water temperatures, but *L. salmonae* was considered a primary pathogen due to the heavy infection and marked associated lesions. However, co-morbidities in the later case involved the primary pathogen *A. salmonicida* (furunculosis), mild trichodiniasis on the gills, and mixed environmental bacterial infections that were all exacerbated by water temperature, low dissolved oxygen, sea lion predation, and delayed maturation. Two additional cases of PSM in wild adult salmon from Alaska involved wild Sockeye Salmon from a remote lake (Shell Lake) and this occurred in both 2012 & 2016 [272,326]. Again, co-morbidities were involved, which included less pathogenic renal myxosporidiosis (*Chloromyxum* sp. and *Sphaerospora* sp.) and heavy *Philonema* sp. burdens in the 2012 case where *L. salmonae* was considered a primary pathogen and cause due to infection level and of lesions. However, *L. salmonae* was likely more of a contributory factor for the later case in 2016 involving severe branchial ceratomyxosis as the primary cause as well as low-level ichthyophoniasis as another secondary disease.

This microsporidium forms xenomas originated in the endothelium of the gills but may be found in other organs, such as the heart, ovaries and kidney. Diagnosis is usually made by wet mounts or histology. The former will reveal typical microsporidian spores with a posterior vacuole, whereas histology is useful for demonstrating xenomas and associated lesions. PCR tests have also been developed [343]. Spores that are released from ruptured xenomas as the infection proceeds are associated with the most tissue damage, as seen in net pen reared salmon [344]. The ability of the spores to resist iodine disinfectants and the occurrence of xenomas in the ovaries [344] suggests a risk of maternal transmission [345]. This is supported by the presence of the parasite in Chile through egg shipments as *Oncorhynchus* spp. [346], the natural host for *L. salmonae*, does not naturally occur in South America.

#### 4.2.3. Minor Myxozoa

##### *Henneguya salminicola* 

Ward described *Henneguya salminicola* from salmon in Alaska [347], whereas a similar species, *H. zschokkei*, was described from Whitefish (*Coregonus* spp.) a bit earlier in Europe. Some parasitologist have considered them to be synonymous, but we follow the designation of Boyce et al. referring to infections in *Oncorhynchus* spp. in the PNW as *H. salmonicola* [348].

Infections are initiated in juveniles and persist throughout their entire life. Species which have extended time in freshwater as juveniles, such as Coho and Sockeye Salmon, tend to be more heavily infected [348]. The life cycle is unknown but based on knowledge from other myxozoans the life cycle probably requires an oligochaete alternate host. Large macroscopic cysts filled with myxozoan spores are found throughout the skeletal muscle [252]. There is minimal tissue reaction to the parasite, and the infection has not been associated with morbidity or mortality in adult or juvenile Pacific salmon species. Hence, the main concern is that the unsightly pseudocysts may reduce the consumer appeal of infected fillets, known by some as ‘milky flesh’ or ‘tapioca’ disease. Yahalomi et al. found that *H. salminicola* has lost aerobic mitochondrial genes [349], whereas they are represented in some other myxozoans.

##### *Kudoa thyrsites* 

The genus *Kudoa* is an assemblage of many species of marine myxozoans that target muscle [350]. *Kudoa thyrsites* is well-known as a cause of post-harvest myoliquefaction and is associated with significant marketing problems in commercial species [350]. It is a common problem in marine net pen reared salmonids in British Columbia, particularly in Atlantic Salmon. In contrast to most reports in somatic muscle [351,352], Kabata and Whitaker reported infections in the cardiac muscle of adult Coho, Chinook, Pink Salmon and Steelhead Trout in rivers in British Columbia [353]. No indication of effects on survival were reported but considering that cardiac muscle is infected [354], perhaps heavy infections may compromise cardiovascular function and subsequently, swimming ability and survival in rivers. Diagnosis can be readily achieved by observing the characteristic stellate spores with four polar capsules in either wet mounts or histologic sections.

##### *Myxidium truttae* 

**Figure 7:**
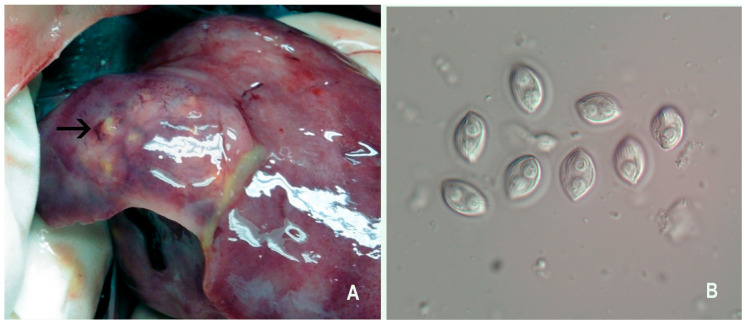
Adult Coho Salmon with *Myxidium truttae* infection. (**A**) Liver with large, multifocal to coalescing regions of pallor and a few, multifocal, yellow to tan nodules (arrow). (**B**) Wet mount of liver showing spores with distinctive bipolar polar capsules and ridges on spore valves suggestive of *M. truttae*.

This myxozoan infects the gallbladder and hepatic bile duct of Brown Trout in Europe [355]. The parasite has been shown to cause jaundice in this host in Italy [356]. Reports of this species occurring in salmonids in North America are rare [252] and to our knowledge there are no records of it causing disease in salmonids from this continent. However, two reports from Alaska demonstrated liver disease consistent with this parasite in Coho Salmon. The first case was in 2016 where a hatchery stock of Coho Salmon from Bear Lake, Alaska (Kenai Peninsula) were submitted for diagnostic investigation of a warm-water associated PSM event with 45% female broodstock that had died s that were dying [357]. The primary cause of mortality was diagnosed as furunculosis, but contributory factors included Gram-negative septicemia, flavobacteriosis, a *Trichodina* infestation, and this hepatic *Myxidium* infection. The liver had macroscopic changes, with an overall mottled appearance and large, multifocal, off-white to yellow foci that were occasionally hemorrhagic in one of two PSM fish. The second case was in 2018 and involved a single wild Coho Salmon that was caught in the sport fishery and displayed grossly visible liver lesions that was the reason for the sample submission. There were no other contributory factors in this case and the fish presented with large, cystic hepatic lesions replete with myxospores [358] (Figure 7A). The parasite was DNA sequenced (NCBI Acc. No. PX480734.1 and PX593113.1) and had a 100% match to *M. truttae* from Europe (NCBI Acc. No. AJ582061.2). As with other myxozoans, diagnosis is readily achieved by observation of characteristics spores. In this case, as with other *Myxidium* spp., the polar capsules are found on opposite ends of the spore, and the spore valves are striated (Figure 7B). This can then be followed by DNA sequencing to confirm the species-level identity.

##### *Myxobolus* sp., *M. arcticus*, *M. cerebralis*, *M. neurobius*, and *M. neurotropus*

Several *Myxobolus* species have been reported from the central nervous system of salmonids (neurotropic myxozoiasis), including the most pathogenic species, *M. cerebralis* [359]. The latter infects vertebrae and other cranial skeletal elements, causing profound deformities, whirling behavior, and high mortality when infections are initiated in juveniles where bones are not completely ossified [360]. Other central nervous system (CNS) species of salmonids (e.g., *M. arcticus*, *M. neurobius*, *M. kistuchi*, *M. neurotropus*) are restricted to central neural tissues and less associated with clinical disease. However, Moles and Heifetz reported that *M. arcticus* was associated with reduced swimming performance in juvenile Sockeye Salmon [361]. Lorz et al. noted a similar *Myxobolus* species to *M. neurotopus* in neural tissues of various juvenile *Oncorhynchus* species in a survey for *M. cerebralis* in Oregon [362].

**Figure 8:**
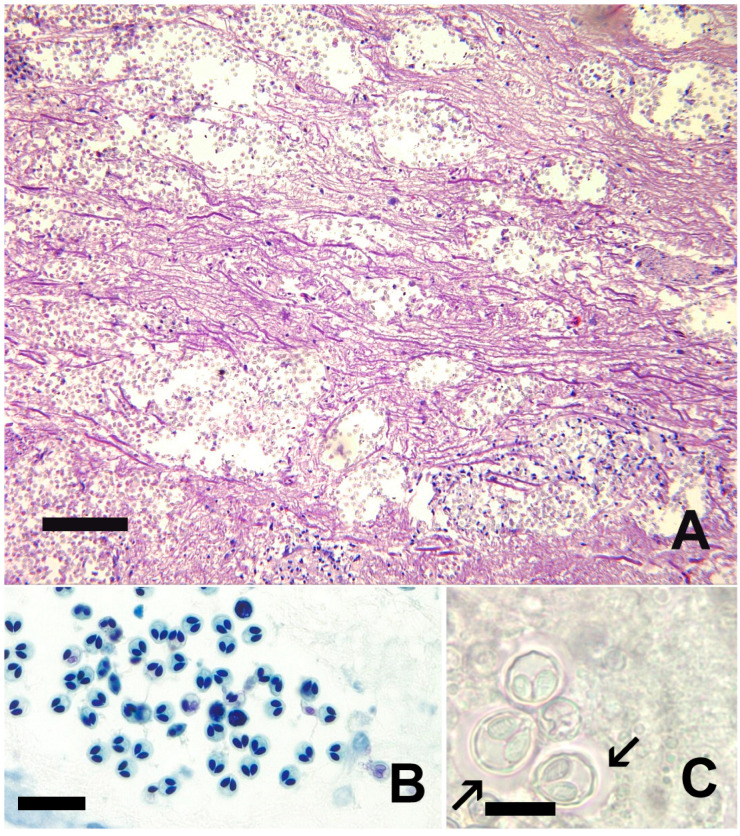
*Myxobolus* sp. From Adult Chinook Salmon central nervous system. (**A**) Dozens of aggregates of spores replacing the neural tissue in the hindbrain. H&E. Bar = 100 µm. (**B**) *Myxobolus* spores with two distinctive polar capsules highlighted in dark blue with a Giemsa stain. Bar = 25 µm. (**C**) Wet mount showing posterior spore envelopes (arrows) and pyriform polar capsules. Bar = 10 µm.

In our survey of hundreds of adult Chinook Salmon in Oregon, the CNS of many fish were infected with a *Myxobolus* sp. [9] (Figure 8). The spores had a similar morphology those of *M. neurotropus*, which has been reported in salmonids in Idaho, Oregon, Washington, California, Utah, and Alaska [363,364]—e.g., subspherical spores with pyriform polar capsules containing 6–8 coils in the polar filament and a mucous envelope at the posterior of some spores. The infection was common within the hindbrain and spinal cord, where large numbers of spores were often present as a focal mass effect, but the infection was associated with minimal inflammation [365]. Hence, damage is mainly caused by the compressive effect of the parasitic mass, leading to loss and replacement of surrounding tissues. This is consistent with many CNS myxozoan infections [365]. Benda et al. reported that the parasite was less frequently observed in Chinook Salmon that had been out planted to the Willamette River than those held through the summer in tanks [9], suggesting that this myxozoan may be associated with PSM in the river. Along with the observations of impaired swimming with *M. arcticus* in Sockeye Salmon, the possibility that the infection seen in adult Chinook Salmon may impair swimming efficiency and thus lead to PSM cannot be excluded. The location and minimal tissue reaction are similar to that seen with neural microsporidiosis in Zebrafish (*Danio rerio*) [366]. The microsporidium causes subtle behavioral changes beyond obvious swimming patterns and efficiency. Perhaps similar behavior changes occur with the *Myxobolus* sp. in adult Chinook Salmon, which could then reduce the survival to spawning in the fall. *Myxobolus arcticus* has also been of importance as it has been used a biologic tag for salmon stock identifications on the high seas [367].

##### *Myxobolus squamalis* 

*Myxobolus squamalis*, first described in 1954 [368], is occasionally observed in the skin of adult salmonids in the PNW, particularly Coho Salmon in freshwater [252,369]. The parasite creates pockets of ovoid spores, with a sutural ridge and four turns of the polar filament within polar capsules [252], expanding the scale pockets of infected fish, causing raised scales and white patches that give an undesirable appearance referred to as “salmon pox” [370]. While most infections are often incidental, heavy infections can cause visible skin lesions that may lead to secondary bacterial or fungal infections, potentially affecting fish market value and health [252]. Diagnosis is based on light microscopy of spores from tissue squashes, histological examination with staining to observe cysts and spores in scale pockets, and molecular methods including PCR amplification [369,370].

Like other myxozoans, *M. squamalis* is presumed to involve an obligate invertebrate host, possibly oligochaete worms, with two waterborne spore stages involved in the life cycle [252,369]. Management strategies for myxozoans, in general, include PCR-based environmental water testing for early detection, water management to reduce spore load in hatcheries, and biosecurity measures to prevent introduction and spread [252]. Currently, there are no documented effective treatments, with management focusing primarily on prevention and early detection [252].

##### *Parvicapsula kabatai* 

*Parvicapsula kabatai* was first described from the kidney of spawning Pink Salmon in the Quinsam River. In Pink Salmon, the annual prevalence of infection was consistently higher when detected by PCR in comparison to histology and between 2003 and 2005, the prevalence ranged from 0% to 69% [371]. The parasite replicates in tubule epithelial cells causing localized distension and necrosis of epithelial cells. Mature spores are released into the lumen. In heavy infections, mature spores may also occur within the interstitium in association with zonal necrosis. The annual prevalence between 2006 and 2013 ranged from 0% to 74.5% (S. Jones, unpublished observations). Between 2003 and 2013, prevalence in Pink Salmon tended to be higher in the odd years (69.2%, 21.7%, 40.0%, 16.7%, 74.3%, 73.3%) compared with the even years (0%, 0%, 8.3%, 3.3%, 15.0%). The parasite was shown by PCR and sequencing of archival kidney samples to occur in marine pen-reared Coho Salmon in Puget Sound [371,372]. The characteristic small spore size and apical location of the polar capsules suggest this was the same parasite reported earlier in pen-reared Coho Salmon from Puget Sound [373,374] and British Columbia [375]. The parasite has also been reported in juvenile Pink Salmon following migration into the ocean [371,376], where it was associated with kidney swelling and diffuse interstitial inflammation. The invertebrate host of *P. kabatai* is not known and the parasite is genetically distinct from *P. minibicornis* and *P. pseudobranchicola*, which also occur in salmonids in western North America [275,371,377].

## 5. Macroparasites

**Table 4:** Review of macroparasite pathogens in adult Pacific Salmon (*Oncorhynchus* spp.) in freshwater from California to Alaska. Major: significant disease or other concerns, and widespread. Minor: restricted outbreaks, although they may cause severe lesions or morbidity.

	Macroparasites	Species Affected	Source (F/M)	System Affected	Disease Description	Location (State or Province)
**MAJOR**	**Copepoda**					
	*Salmincola californiensis*	All	F	Gi	Asymptomatic, respiratory compromise, branchitis	CA, OR, WA, BC, AK
	**Trematoda**					
	*Nanophyetus salmincola*	All	F	Sys, V, R, M, Gi	Systemic tissue cysts; salmon poisoning in dogs	CA, OR, WA, BC, AK
**MINOR**	**Arthropoda**					
	*Lepeophtheirus salmonis*	SO	M	I	Erosive dermatitis	CA, OR, WA, BC, AK
	**Cestoda**					
	*Dibothriocephalus* spp.	All	M	M, P, C	Tissue metacestodes (zoonotic risk)	CA, OR, WA, BC, AK
	*Dibothriocephalus nihonkaiense*	PI	M	M	Tissue metacestodes (zoonotic risk)	AK
	*Dibothriocephalus ursi*	SO	M	G	Gastric metacestodes (zoonotic risk)	AK
	*Diphyllobothrium cordiceps* (syn. *D. dendriticum*)	SO	M	P	Peritoneal metacestodes (zoonotic risk)	BC
	*Eubothrium* sp.	All	M	G	Gastroenteric metacestodes, juvenile SO morbidity	CA, OR, WA, BC, AK
	*Pelichnibothrium* sp. ‘larva’	CH	M	G	Asymptomatic, enteritis	OR, WA
	**Nematoda**					
	*Anisakis* spp. larvae	All	M	M, V, P	Tissue cysts (zoonotic risk)	worldwide
	*Philonema* spp. (*P. agubernaculum* and *P. oncorhynchi*)	All	M	P	Tissue cysts	BC, AK
	**Trematoda**					
	*Apophallus* sp.	CH	F	G, Gi	Asymptomatic, cartilage metacercariae	OR
	*Echinochasmus milvi*	CH	F	G, Gi	Asymptomatic, cartilage metacercariae	OR

Species affected: Sockeye (SO), Chinook (CH), Chum (CU), Coho (CO), Steelhead (SH), Pink (PI). Source of infection: M = marine and/or F = freshwater. System affected: G = gastrointestinal, I = integument, R = renal, M = musculoskeletal, P = peritoneal, C = cardiovascular, V = viscera, Gi = gill.

Macroparasites, as defined by Brown, are larger, multicellular organisms that do not multiply directly within their vertebrate hosts [206]. This section focuses on the trematodes, nematodes, cestodes, and arthropods that parasitize adult Pacific salmon during their spawning migrations (Table 4). There are several surveys and reviews that list countless macroparasites in salmonids [21,22,378,379,380]. Most of these reports by parasitologists focused on parasite characteristics and did not note associated pathologic changes. See our summary data at ScholarsArchive@OSU (https://ir.library.oregonstate.edu/concern/datasets/st74d079z [accessed on 10 January 2026]). Major macroparasites as we define them include the trematode *Nanophyetus salmincola*, and the copepod *Salmincola californiensis*, which can both significantly impact salmon health and survival through tissue damage, organ dysfunction, decreased metabolic status, and secondary infections. Minor macroparasites include Sea Lice (*Lepeophtheirus salmonis*), various nematodes (*Anisakis* spp., *Philonema* spp.), cestode larvae (including *Dibothriocephalus* spp.), and additional trematode metacarcariae that we noted in the gills of Chinook Salmon.

The macroparasites of adult spawning Pacific salmon exhibit diverse life cycles, often involving multiple hosts and complex transmission dynamics between marine and freshwater environments. Infection intensity varies widely among individual fish and salmon species, with some parasites showing strong host preferences. Many of these parasites affect specific organ systems, including the gastrointestinal tract, musculature, reproductive organs, and gills, with varying pathological consequences. Some, like *N. salmincola* and *Anisakis* spp., have additional significance as vectors for other pathogens or direct pathogens as zoonotic agents, with the former being associated with mortality in juvenile Pacific salmon.

### 5.1. Major Macroparasites

#### 5.1.1. Copepoda

##### *Salmincola californiensis* 

*Salmincola californiensis*, often called gill maggots, infects various *Oncorhynchus* species, including Chinook, Sockeye, and Coho Salmon and Steelhead and Rainbow Trout [381,382]. It infects the gills of salmonids in freshwater throughout the PNW. In Oregon, the parasite can cause lethal infections in juvenile salmon, particularly in out-migrating Chinook Salmon smolts in reservoirs above dams [383,384]. Laboratory studies confirmed the lethality of the parasite [385]. In addition, the infection causes profound reduction in swimming stamina [386]. Wet mounts reveal massive removal of gill tissue, including areas of primary filaments around parasites due to feeding activity, resulting in focal truncation of filaments [385,387]. Histology reveals chronic, focal inflammation at the attachment location of the bulla [388]. Whereas the copepod is quite detrimental to juvenile fish, it is common in adult Chinook Salmon without obvious clinical disease. Infections can be very intense, but most parasites, represented by gravid females, are found toward the tips of the gills in adult salmon [117]. Although not clearly documented to cause significant mortality in most captive adult salmon, concerns with the infection include these fish being reservoirs for infections to out-migrating smolts and transmission of *A. salmonicida* [117].

Diagnosis is achieved by macroscopic observation of females with egg sacs attached to the gills, the inner opercular lining, or less commonly the base of the fins. Copepodids and calamus stages are smaller and can be visualized in microscopically wet mounts [387]. If needed, differential diagnosis between *S. californiensis* and *Salmincola edwardsii*, the latter of which occurs more toward eastern North America, can be achieved by examination of parasite appendages or molecular methods [388].

Captive fish can be treated with ivermectin gavage at 0.2 mg/kg fish [389,390]. Reed treated adult spring Chinook Salmon and adult Rainbow Trout with doramectin at 0.2–0.4 mg/kg by intramuscular injection that reduced infections [391]. The treatment prevented reinfection on Chinook Salmon for 8 weeks post injection and decreased the PSM in the trout from 30% in the control group to 0% in the treated fish.

#### 5.1.2. Trematoda

##### *Nanophyetus salmincola* (Salmon Poisoning)

*Nanophyetus salmincola* is a common metacercarial infection in fishes in the PNW from northern California through Washington State. Impacts are essentially limited to juvenile salmonids [392,393,394]. However, it is considered a Major Pathogen as the worm is host to *Neorickettisia salmonisitica*, the cause of a serious systemic disease of the Dog (*Canis familiaris*), called Salmon Poisoning [395]. The range of infection in salmon is dictated by the geographic range of the Pleated Juga Snail (*Juga plicifera*), the first intermediate host, which includes western regions of Northern California north through Washington State. Salmonids and other freshwater fishes are the second intermediate hosts, in which metacercariae encyst in various organs, including gills, muscle, kidney and visceral organs. Definitive hosts are mammals that consume live metacercariae; bears, otters, raccoons, dogs, and occasionally humans [395].

**Figure 9:**
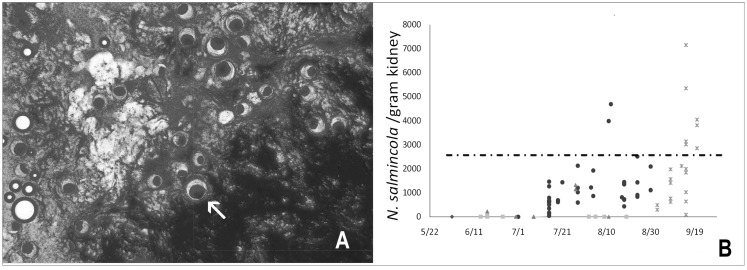
*Nanophyetus salmonicola* metacercariae in the kidney of an Adult Chinook Salmon. (**A**) Wet mount of kidney with numerous metacercariae. A diagnostic feature is the presence of a posterior, opaque spherical structure representing the excretory vesicle (arrow). Black background is due to extensive melanin deposits in renal interstitium. (**B**) Increase density of metacercariae in kidneys of adult salmon from the Willamette River through the summer of 2010. *X*-axis includes month and day collection. Black Diamond = Live caught down early in the run at Willamette Falls, Grey Square = Live caught from Fall Creek. Triangle = live caught midsummer and midriver at Dexter Dam. Black Circle = PSM, dead collected from river. X = artificially spawned fish at Willamette Hatchery in the fall. Horizontal dashed line = prediction of level of parasite associated mortality using Crofton’s truncation model of the negative binomial distribution [393,398].

Encysted *N. salmincola* metacercariae in fish are spherical, about 100–200 µm. They are distinguished from other metacercaria by a prominent posterior excretory vesicle, which appears dark in squash wet mount preparations (Figure 9A). Parasite associated mortality has been linked to increased burdens in juvenile Coho Salmon and subclinical disease of reduced swimming performance, growth, and smoltification [394] and direct mortality [396], but to date this has not been directly correlated with PSM in adult salmon. Benda et al. noted that the parasite was not linked specifically to PSM in spring Chinook Salmon from Oregon [9], which was further supported by personal communication with R. Holt, ODF&W. Nevertheless, burdens increase in adult salmon that survive to adulthood after they return to freshwater, probably due to new infections (Figure 9B). Indeed, by the end of the summer many spring Chinook Salmon in Oregon have >1000 metacercaria/g in kidney. Similar increases as time progresses have been reported in Chinook Salmon from California [397].

The infection causes the most impact in domestic dogs and other canids that ingest the metacercariae, resulting in the life-threatening disease Salmon Poisoning [395]. Clinical disease is caused by *Neorickettsia helminthoeca*, an obligate intra-cytoplasmic bacterium, for which the trematode is a vector. This results in a fatal septicemia if infected dogs are not quickly treated with antibiotics due to hemorrhagic enteritis. A novel neorickettisal bacterium, the *Stellanchasmus falcatus* (SF) agent, also causes gastrointestinal disease in dogs, sometimes manifesting with a similar clinical profile as Salmon Poisoning [399]. We detected SF in *N. salmincola* from Chinook Salmon adults [400]. The infection is essentially impossible to avoid in adult fish in endemic areas, hence the infection in dogs is controlled by preventing them from eating raw, adult salmon, prompt diagnosis of the disease, and treatment with antibiotics. Most, but not all, dogs develop protective immunity if they survive the bacterial disease. However, the reason that some dogs develop Salmon Poisoning more than once is unknown—i.e., an inadequate immune response or infection by a different strain or species (e.g., the SF agent).

### 5.2. Minor Macroparasites

#### 5.2.1. Arthropoda

##### *Lepeophtheirus salmonis* 

*Lepeophtheirus salmonis*, colloquially called Sea Lice, is a common ectoparasitic copepod affecting salmonids in seawater, with debates regarding its impacts on survival in wild fish [401]. While primarily a concern in seawater net pen-reared salmon, significant infections were documented in wild adult Sockeye Salmon [402]. There were reported severe skin lesions characterized by extensive erosion and hemorrhage and high mortality in adult Sockeye Salmon returning to Barkley Sound, British Columbia, particularly in 1990, with less severe lesions observed in 1992 and 1993. The authors concluded that PSM likely occurred because while a high percentage of fish had open skin lesions in marine waters, few fish with such lesions were observed on the spawning grounds.

#### 5.2.2. Cestoda

Tapeworms (cestodes) are often observed as adults in the gastrointestinal tract of salmonids (e.g., *Eubothrium* spp., *Proteocephalus* spp., *Cyathocephalus truncatus*, *Bothrimonus olrikii*) or as metacestodes/plerocercoids (larval/immature stages) in the intestine, viscera and musculature (*Dibothriocephalus* spp.; *Trypanorhyncha*, especially *Nybelina* sp.) [356,379,403]. Occasionally, metacestodes of trypanorhynchs can infect unusual sites, as in the case of an infection by *Gilquinia squali*, a tapeworm of sharks, in the eyes of pen-reared Chinook Salmon [404]. Although these tapeworm infections in salmonids can cause localized histopathologic changes, especially at sites of attachment in the intestinal tract [405,406], they are not often associated with significant tissue damage or PSM. Exceptions include metacestodes of *Triaenophorus crassus* in Whitefish [407,408,409,410] and in (experimentally infected) Rainbow Trout [410]. However, certain diphyllobothriid (Family Diphyllobothriidae) species (e.g., certain *Dibothriocephalus* spp.) utilize salmonids (and other fish) as intermediate hosts to infect humans [411] and infections can be unsettling to anglers.

##### *Diphyllobothrium*/*Dibothriocephalus* spp.

The tapeworm family *Diphyllobothriidae* contains the commonly named ‘broad tapeworms’, of which certain species in the genus *Dibothriocephalus* (formerly *Diphyllobothrium*) remain important for their ability to infect humans and because some use salmonids as intermediate hosts to do so [411,412,413,414,415]. Of these, *D. ditremus*, *D. dendriticus*, *D. ursi*, *D. nihonkaiense*, and *D. nihonkaiensis* are found in salmonids as plerocercoid metacestode stages that are infective to the final (definitive) hosts where these tapeworms mature and reproduce.

The basic life cycle of *Dibothriocephalus* ‘broad tapeworms’ is similar in having a copepod first intermediate host and a fish second intermediate host. The final (definitive) hosts are fish-eating birds or mammals. Each species of *Dibothriocephalus* has its own typical definitive host or small group of such hosts; *D. dendriticus* and *D. ditremus* are most often found in gulls (Laridae) but can infect other fish-eating birds such as pelicans (*Pelecanus* spp.), whereas *D. ursi* and *D. nihonkaiensis* typically infect Bears (family Ursidae) as definitive hosts. The iconic human broad tapeworm, *D. latus* (=*Diphyllobothrium latum*) does not use salmonids as intermediate hosts in its circumboreal native range; in North America, its plerocercoid stages are typically found in the flesh of Northern Pike, Yellow Perch (*Perca flavescens*), Walleye (*Sander canadensis*) and possibly Burbot (*Lota lota*). Curiously, it infects introduced and naturalized *Oncorhynchus* species in Chile and Argentina [416,417,418] where human infections apparently sustain the life cycle. Canids (Dogs and foxes (family Canidae)) can also be suitable hosts of *D. dendriticus*, *D. nihonkaiensis*, and *D. latus*. It is important to mention that Pacific records of *D. latus* observations may be misidentified, and this parasite is currently considered limited to European geographic distribution [419].

Pacific salmon (*Oncorhynchus*) species can be second intermediate hosts to the plerocercoid metacestode stages of *D. ditremus*, *D. dendriticus*, *D. ursi*, *D. nihonkaiense*, and *D. nihonkaiensis*, and *D. ditremum* [413,420,421]. Of these, *D. nihonkaiensis*, a well-known species in Japan, and was reported for the first time in North America by Kuchta et al. who found plerocercoids in Pink Salmon in Alaska [420]. Of note is that a massive infestation of *D. ditremum* in Coho Salmon rearing free in a one-way barriered lake for an enhancement program in Alaska caused clinical disease involving liver failure, blood loss, osmotic imbalance and high mortality [422]. Perhaps this lake environment allowed for unusually high abundance of copepods infected with the first intermediate host, which led to high levels in forage fish that were consumed by the salmon.

##### *Eubothrium* spp.

The genus *Eubothrium* comprises nine or ten recognized species of tapeworms that infect the intestinal tracts of marine, anadromous and freshwater fishes [423,424,425]. Two species, *E. salvelini* and *E. crassum*, are typically found as adults in the ceca and intestines of salmonid fishes [356,379,403,426]. Of these, *E. salvelini* appears to be typical of chars, *Salvelinus* spp. (usually Arctic Charr and Lake Trout, *S. namaycush*). In contrast, *E. crassum* is more typical of Pacific salmon (*Oncorhynchus* spp.) in North America, Atlantic Salmon, and Brown Trout in Europe [356,424,426,427,428]. However, both species can infect other salmonids outside their typical hosts and even the same salmonid host simultaneously [428].

Mature *E. crassum* and *E. salvelini* can be differentiated by the number of notches (grooves or incisions) on the apical discs of their scoleces, the width of their neck, position of vitelline follicles and egg size, among other features [426,428]. The life cycle of *Eubothrium* spp. involves a copepod first intermediate host and while transport/paratenic hosts may be involved, ingestion of copepods containing the larval stage(s) by appropriate salmonid hosts is sufficient for the infection. Curiously, there is a marine variant of *Eubothrium*, similar to *E. crassum*, that infects anadromous salmonids in their marine phase and is brought into freshwater environments by returning salmonids on their spawning run. The precise identity of this marine form remains to be determined. *Eubothrium* tapeworms observed by us in returning Chinook Salmon in Oregon (unpublished observations) are likely this marine form because adult *Oncorhynchus* spp. cease feeding after returning to freshwater, which precludes freshwater transmission of these tapeworms.

Most of the information on the effects of infection with *E. crassum* comes from studies on salmonids (mainly Atlantic Salmon) in Europe, including farmed salmonids [429,430,431]. The effects of *E. crassum* on natural populations of *Oncorhynchus* spp. remain largely unknown. Experimental infections of Sockeye Salmon fry with *E. salvelini* resulted in impaired swimming, reduced growth and increased mortality through to the smolt stage [432]. Other effects of *E. salvelini* included impaired seawater adaptation and lowered resistance to zinc in Sockeye Salmon yearlings and juveniles, respectively [433,434].

##### *Pelichnibothrium* sp. *metacestodes* (Immature Post-Larval Stages)

**Figure 10:**
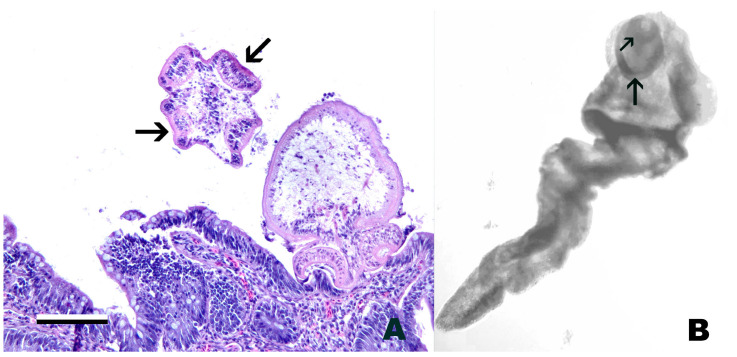
Pleurocercoid stage of *Pelichnibothrium* sp. (Cestoda) in the intestine of captive spawning Chinook Salmon. (**A**) Histological section showing portions of two worms, one free in the lumen showing the arrangement of four suckers (arrows), as well as attached to the intestinal mucosa. H&E. Bar = 100 µm. (**B**) Whole worm from the intestine (frozen and thawed for necropsy). Large arrows point to the bothridia (suckers), small arrow points to an accessory sucker.

Metacestode stages of tapeworms consistent in morphology with *Pelichnibothrium* were observed in the intestinal lumen of adult Chinook Salmon in both Washington and Oregon (Figure 10). *Pelichnibothrium* is a marine tapeworm genus, and salmonids are atypical hosts where immature stages are found in the intestine. The scolex bears an apical sucker and four broadly attached bothridia, each with a small anterior, marginal accessory sucker [435]. Hoffman lists larval stages of *Pelichnibothrium* from all five Pacific *Oncorhynchus* species (Chinook, Chum, Coho, Pink, and Sockeye Salmon) [356].

There are reportedly two species in this tapeworm genus, of which one, *Pelichnibothrium speciosum*, is a well-described, valid species. It is a widely distributed parasite as adults in the Blue Shark (*Prionace glauca*), and the typical intermediate fish host is the widely distributed Longnose Lancetfish (*Alepisaurus ferox*). Larvae have been reported from anadromous salmonids as well, but based on morphology only [435,436]. The other species, *P. caudatum* was described in 1914 by Zschokke and Heitz based on metacestodes from Chum Salmon in Kamchatka [437]. The adult is unknown, and the species has been considered a “*species inquirenda*” (of doubtful identity needing further investigation). The occurrence of metacestodes of this marine tapeworm in our surveys of adult Chinook Salmon in Oregon and Washington, influencing freshwaters suggests that following infection in the ocean, perhaps from ingesting a paratenic host, the worm can remain in this immature metacestode state in the intestinal lumen for many months.

The identity and taxonomic status of these metacestodes of *Pelichnibothrium* require verification using molecular methods because they resemble other phyllobothriid metacestodes, such as *Phyllobothrium salmonis*, that have also been reported from Pacific salmonids. Yamaguti also considered *P. salmonis* a synonym of *Pelichnibothrium speciosum* [436].

#### 5.2.3. Nematoda

##### *Anasakis* spp. *larvae*

*Anisakid larvae* (Fam. Aniskaidae, e.g., *Anisakis* spp. and *Phocanema* spp.) are common nematode parasites of many marine fishes around the world, including Pacific salmon throughout their North Pacific range [438]. Fish serve as intermediate hosts, where third-stage larvae (L3) are ingested by marine mammals to complete their life cycles. Likewise, humans can become infected if they eat raw or improperly cooked fish, and at times these infections can cause complications when larval worms penetrate the stomach or intestine. There are also reports of humans developing an allergy to the worm [439,440]. These parasites have been documented in all five species of Pacific salmon, with high prevalence rates reported in studies from Alaska and the PNW. Karl et al. found infection rates of up to 100% in some samples of Alaskan Sockeye, Pink, and Chum Salmon [441], while Deardorff and Kent reported 100% infection by *A. simplex* in Sockeye Salmon in Washington State [442]. Interestingly, a study using canned salmon that were caught across a broad range in Alaska from 1979–2019 showed infections in Chum and Pink Salmon significantly increased over this 42-year period and may have been associated with the implementation of the Marine Protection Act of 1972 resulting in an increase in definitive host species populations [443].

The larvae primarily encyst in the visceral muscle, viscera, and ventral abdominal wall of infected fish, with lower frequencies in the tail muscle and other parts [441]. The distribution within the fish is not uniform, with the highest concentration typically found in the visceral muscle and viscera. Deardorff and Kent noted that the larvae can be found throughout the musculature and viscera of infected salmon [442]. With infections usually focused on mesenteries and muscle, fish can tolerate heavy infections. They may occasionally penetrate through the intestinal layers [444], but usually associated pathologic changes are localized around worms [445]. We frequently observed worms along the serosal lining in histologic sections of the intestines in our multi-year adult Chinook Salmon survey in Oregon.

While *Anisakis* infections cause no apparent clinical signs in adult salmon [442], the presence of these parasites is primarily a concern for human health rather than fish health through zoonotic infections. The larvae pose a risk to humans consuming raw or undercooked fish, potentially causing severe gastrointestinal symptoms. Hence, from a salmon fisheries management perspective, *Anisakis* infections are primarily a food safety concern rather than a significant threat to salmon health during spawning migrations. Heavy infestations are also unsightly and can impact fisheries due to product quality issues.

##### *Philonema* spp.

Dracunculoid nematodes of the genus *Philonema* are commonly found as subadults and adults in the abdominal cavity of salmonid fishes. They are slender (filiform), soft-bodied, flaccid, translucent worms. Two, morphologically similar, species, *P. oncorhynchi* and *P. agubernaculum*, occur in North American Pacific salmon [446,447,448]. The two species can be differentiated by molecular methods [446,449] and different life cycle characteristics [448,450]. *P. oncorhynchi* occurs as gravid adults in anadromous salmon returning to spawn, whereas *P. agubernaculum* reportedly only matures in resident, freshwater salmonids (mainly *O. mykiss*). While *P. oncorhynchi* reportedly only infects juveniles of anadromous salmon species [451], *P. agubernaculum* can infect juveniles of both anadromous and freshwater resident *Oncorhynchus* species [446,448,450].

The basic life cycle of both *Philonema* species is similar and completed in freshwater; gravid females in the abdominal cavity of their salmon hosts release first-stage larvae (L1) that are likely voided with the hosts’ eggs during spawning before spawning eggs are released into the coelomic cavity and then expelled through a pore adjacent to the anus. L1 larvae, now free in the water, are ingested by copepods, where they molt and develop to the infective L3 larvae. When juvenile salmon ingest copepods with L3 larvae, the larvae evidently penetrate the gut of their juvenile salmon hosts and establish in the abdominal cavity where they grow and reach sexual maturity. The presence of adult *P. oncorhynchi* in returning anadromous salmon suggests that adults can be long-lived.

*Philonema* infections are not generally associated with notable disease in wild Pacific salmon. Heavy infections can result in visceral adhesion [452,453,454], but research on the effects of the parasite on wild salmon produced mixed results. Garnick and Margolis suggested that *Philonema* infections could affect the orientation of seaward migrating salmon smolts [452]. Nagasawa concluded that visceral adhesions caused by *P. oncorhynchi* infections had no effect on the growth of Sockeye Salmon in the North Pacific and Bering Sea [455]. Berg et al. found that even heavy infections of *P. oncorhynchi* in Alaskan Sockeye Salmon did not reduce fish mass/length nor did it affect sexually dimorphic traits under selection [456]. A recent study indicated that *P. oncorhynchi* infections could potentially cause mortality in juvenile Chinook Salmon in an Oregon reservoir although the authors called for more studies [451]. In contrast, disease appears to be more common in certain aquaculture situations. Yearling Coho Salmon and Steelhead reared in freshwater net-pens and that were also heavily infected with *P. agubernaculum* presented abdominal distension with ascitic fluids [457]. Brocklebank et al. reported diffuse granulomatous enteritis, splenitis, pancreatitis, and steatitis with marked fibrosis associated with long-standing infections of *P. oncorhynchi* in farmed Coho Salmon smolts and post-smolts [458]. *Philonema* was listed as the primary pathogen in PSM in Sockeye Salmon from two locations in British Columbia (BCFHD cases 2012017 and 009209). It was also associated with severe disease involving visceral adhesions, granulomatous inflammation with marked fibrosis, and caseous necrosis in an adult Coho Salmon from Alaska in two separate cases [459,460]. We assume, like many parasites that the most important factor in severity of disease is based on abundance of worms, followed by premature development of the worms as apparently occurred in immature salmon [461].

#### 5.2.4. Trematoda (Metacercariae)

**Figure 11:**
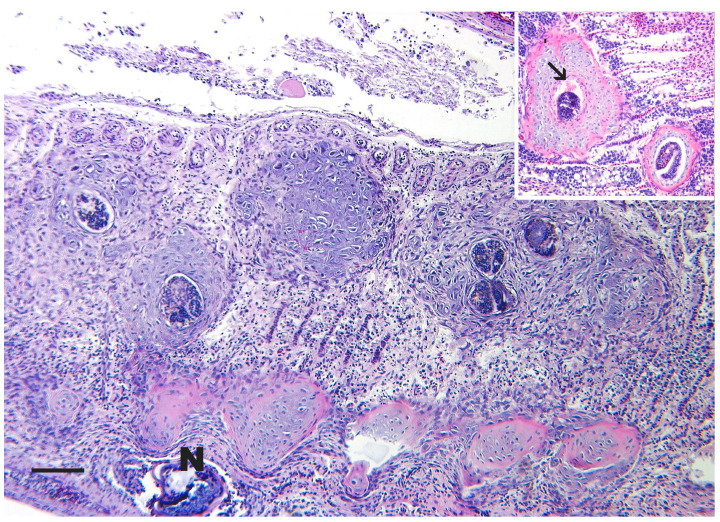
*Metacercariae* in gill tissue of an adult Chinook Salmon surrounded by prominent cartilaginous proliferation. N = large metacercaria with excretory vesicle consistent with *Nanophyetus salmonicola.* Bar = 100 µm. Insert shows a metacercaria consistent with the genus *Echinochasmus* with oral collar spines (arrow). Worm on right is more consistent with *Apophallus* sp. H&E.

In addition to *N. salmincola* (a Major parasite), several species of trematodes infect salmonid fishes as both metacercariae and adults (https://ir.library.oregonstate.edu/concern/datasets/st74d079z [accessed on 10 January 2026]). The latter generally infects the lumen of the gastrointestinal tract and are thus relatively non-pathogenic if not in excessively high numbers. In contrast, given the location of *Metacercariae* deep within tissues, this stage is generally considered more pathogenic than adult worms with several studies showing this in juvenile salmon. For example, *Apophallus* sp., later described as *Apophallus microsoma* [462]. Regarding adult fish, two types, presumably belonging to two or three species, were of note in our histologic survey of adult Chinook Salmon in Oregon [393,463]. Our survey of gills from 576 fish revealed 69% infected with *Metacercariae* extending through the cartilage of primary gill filaments (Figure 11). *Metacercariae* with oral spines were putatively identified as *Echinochamus milvi* and others without obvious spines as *Apophallus* sp. [464]. Their role in morbidity and PSM was not determined, but given the extent of the lesions the gill function may be compromised when respiratory demands are high, such as swimming against strong river currents. Similar lesions with profound cartilaginous proliferation are caused by *Metacercariae* of *Centrocecus formosanus* [465] and have been associated with morbidity. Nevertheless, linking these infections to morbidity in adult Chinook Salmon is presently only speculative.

## 6. Discussion

Adult *Oncorhynchus* species are documented to be hosts for a long list of pathogens. We designated only one virus (IHNV) as a major concern due to maternal transmission as this pathogen results in high mortality of progeny. However, several bacteria were assigned to this category. A remarkable number of parasites have been recorded from salmonid fishes [21,378,379,380,403], where they have caused varying levels of mortality in juveniles, some quite high, but most have not been associated with adult PSM. In this review, we designate nine parasites as Major and over 20 as Minor. However, countless other parasites have been described from salmonids in the PNW for which their pathogenic potential is unknown (see https://ir.library.oregonstate.edu/concern/datasets/st74d079z [accessed on 10 January 2026]). Adult salmon in freshwater rivers are often subjected to severe extrinsic stressors such as low dissolved oxygen, elevated water temperatures, other poor water quality parameters, and obstruction of their upriver migrations. Anthropogenic stressors, such as harassment in spawning areas [103] or chemical contaminates are also a concern [5]. In addition, these fish are immune compromised due to natural, life-stage specific physiologic processes [11,12]. Hence, fish at this stage in their life are particularly susceptible to new freshwater infections, reactivation of latent pathogens, or expansion of pre-existing infections that may manifest as disease. This leads to multiple concurrent infections, making it often difficult to link specific pathogens or syndromes to PSM [9], as we have seen with ASE in Chinook Salmon [10,320]. Negative synergistic interactions of multiple etiologies should be considered [466]. Moving to the future, with climate changes leading to warmer water and reduced water flow [467], it is likely that virulence of certain recognized pathogens in adult salmon will increase and/or increased host susceptibility disease.

Diseases of salmonid fishes in the PNW have been studied for decades, but most efforts have been directed to those afflicting juvenile fish in hatcheries. Hence, whereas our review of pathogens in adult *Oncorhynchus* species is extensive, the possibility of novel pathogens as significant contributors to PSM should be considered. For example, we recently described a novel enteric virus from fish with ASE [320]. Given the large variety of documented pathogens, and the potential for novel ones, we recommend that histopathology be included with investigations attempting to link pathogens to PSM as it does not rely on a priori list of agents. Indeed, this method discovered ASE [10]. Histology may be prohibitively expensive for processing multiple organs from large numbers of fish. Hence, a strategy that we often use is to collect comprehensive samples and archive them in formalin. Then a subset is processed and evaluated to guide more targeted processing with the large group of samples. Meyers and Hickey provide guidance on diagnostic approaches to link the presence of a pathogen or its genetic material with repeatedly demonstratable and definable disease [23,468], which is particularly important nowadays in which environmental DNA or host infection surveys are often conducted using only molecular methods. Another useful approach is conducting controlled laboratory in vivo transmission experiments to clarify links of pathogens with disease [17,92,250,314,320,469].

There is now a movement to remove dams on important salmon runs to improve water quality and access to historic spawning grounds. However, one issue to consider is exposure of stocks to novel species or strains of pathogens or ones that the population has not encountered before, or at least for many decades [470]. The same applies to a long-standing practice of volitional passage or trapping and hauling of adult salmon over high head dams so that fish have access to upstream spawning habitats [471]. Hopefully our review here will aid in future monitoring efforts and investigations of PSM in salmon in the Pacific Northwest.

## 7. Conclusions

From this extensive review of salmonid pathogens, we surmise that each species, geographic location, and watershed has varying severity of disease associated with pathogens. Moreover, this severity often varies between years. Whereas some outbreaks of PSM have been clearly linked to specific pathogens, many others are multifactorial and associated with multiple pathogens. Based on our experience, that of other salmon health experts, and reports and peer-reviewed literature, we can conclude that across host species and areas, the most important pathogens are bacteria (i.e., *Aeromonas salmonicida*, *R. salmoninarum*, *Flavobacterium* spp.) and *Saprolegnia* spp. It should still be recognized, however, that some regions have additional major problems—e.g., *Ichthyophonus* spp. in Alaska, ASE and *C. shasta* in Oregon, and *I. multifiliis* in California. Regarding management, there is no “one size fits all”, but, in general, we can recommend (1) having an adequate understanding of the pathogens in normal and PSM fish for a given population and outbreak, (2) attempt to elucidate links to these pathogens and disease and moribundity, and (3) identify factors that are exacerbating these infections and associated diseases. Corrective management options are often limited, but may include avoiding infections that have limited distribution, reducing impact of ubiquitous opportunists by improving water quality, handling and holding conditions, and in some cases targeted chemotherapy. In addition, climate change-associated trends of reduced water flows and elevated temperatures in many watersheds in this region, are likely to exacerbate the impacts of pathogen infections. Our review has led us to conclude that there are still many knowledge gaps in discerning primary causes of disease and PSM given the challenges that adult salmon face in freshwater and their natural immunocompromised state. However, accepting these complexities, we encourage continued research to disentangle and prioritize factors leading to PSM.

## Data Availability

The original data presented in the study are openly available in the ScholarsArchive@OSU at Oregon State University at https://ir.library.oregonstate.edu/concern/datasets/st74d079z (accessed on 10 January 2026). Case report contact information for Alaska: Alaska Department of Fish & Game, Division of Commercial Fisheries, Fish Pathology Section, 333 Raspberry Rd., Anchorage, AK 99515 USA—2025 Contact: Jayde Ferguson, Fish Pathologist. jayde.ferguson@alaska.gov. Case report contact information for California: California-Nevada Fish Health Center, U.S. Fish and Wildlife Service, 24411 Coleman Hatchery Road, Anderson, CA 96007, USA—2025 Contact: Ron Stone, Project Leader. ron_stone@fws.gov.

## Abbreviations

The following abbreviations are used in this manuscript:

ASE	adult salmon enteritis
AIDS	acquired immunodeficiency syndrome
BADI	bilateral acute depigmentation of iris
BAIT	bilateral acute iris transillumination
BKD	bacterial kidney disease
BCFHD	British Columbia Fish Health Database
BCWD	bacterial coldwater disease
CHSE-214	Chinook Salmon embryo
CFU	colony-forming units
CNS	central nervous system
CTV	Cutthroat Trout virus
DFO-PBS	Fisheries and Oceans Canada, Pacific Biological Station
DNA	deoxyribonucleic acid
EIBS	erythrocytic inclusion body syndrome
EPC	epithelioma papulosum cyprinid
ELISA	enzyme-linked immunoabsorbent assay
FAT	fluorescent antibody testing
FPM-A	Flavobacterium psychrophilum medium A
GMS	Grocott–Gömöri’s methenamine silver
IHC	immunohistochemistry
IHN	infectious hematopoietic necrosis
IHNV	infectious hematopoietic necrosis virus
INADs	investigational new animal drugs
IPNV	infectious pancreatic necrosis virus
ISH	in situ hybridization
L1	first-stage
L3	third-stage
mL	milliliter
NWHS	National Wildfish Health Survey
NCBI	National Center for Biotechnology Information
ODF&W	Oregon Department of Fish & Wildlife
ppm	part per million
PAS	periodic acid-Schiff
PCR	polymerase chain reaction
PFU	plaque-forming units
PKD	proliferative kidney disease
PNW	Pacific Northwest
PRV	piscine orthoreovirus
PSM	prespawn mortality
qPCR	quantitative polymerase chain reaction
SFV	salmon flavivirus
SSU	small-subunit
SRS	salmonid rickettsial septicemia
TYES	tryptic yeast extract serum
USDA	United States Food and Drug Administration
WOAH	World Organisation for Animal Health
VENV	viral erythrocytic necrosis virus
VHSV	viral hemorrhagic septicemia virus
